# Sensory processing sensitivity is associated with state-dependent stabilization of perceptual organization in auditory streaming

**DOI:** 10.1038/s44271-026-00482-z

**Published:** 2026-06-09

**Authors:** Hirohito M. Kondo, Daniel Pressnitzer

**Affiliations:** 1 School of Psychology, Chukyo University, Nagoya, Aichi Japan; 2 Laboratoire des systèmes perceptifs, Département d’études cognitives, École normale supérieure, PSL University, CNRS, Paris, France

**Keywords:** Psychology, Human behaviour

## Abstract

Perceptual experience is shaped not only by external input but also by stable individual traits. Here, we investigated how sensory processing sensitivity, a personality trait reflecting responsiveness to sensory input, relates to the temporal dynamics of bistable auditory streaming. Forty-eight participants listened to repeating triplet-tone sequences and continuously reported their percepts as they switched between an integrated one-stream percept and a segregated two-stream percept. Greater sensory processing sensitivity was reliably associated with longer percept durations in the two-stream state and fewer perceptual switches overall. A Bayesian beta-regression model provided little evidence that this pattern reflected a generic bias toward segregation or a global slowing of perceptual dynamics. In contrast, a time-resolved discrete-time hazard model indicated that greater sensory processing sensitivity was linked to a lower termination probability for the two-stream percept relative to the one-stream percept. A Gamma distributional regression further showed that trait-related differences were expressed mainly as changes in dominance-duration timescale, whereas effects on distributional regularity were modest. Thus, sensory processing sensitivity was associated with the temporal dynamics of auditory streaming mostly through the stabilization of perceptual segregation once established. This state-dependent pattern is broadly compatible with a predictive-processing framework, in which stronger weighting of temporal regularity cues could stabilize an already established perceptual organization.

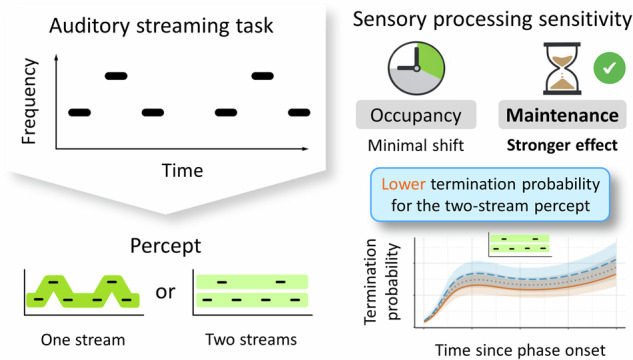

## Abstract (web summary)

Using continuous reports during auditory streaming, this study shows that higher sensory processing sensitivity is linked to stronger maintenance of perceptual organization, suggesting trait-related modulation of auditory scene analysis.

## Introduction

**Fig. 1:**
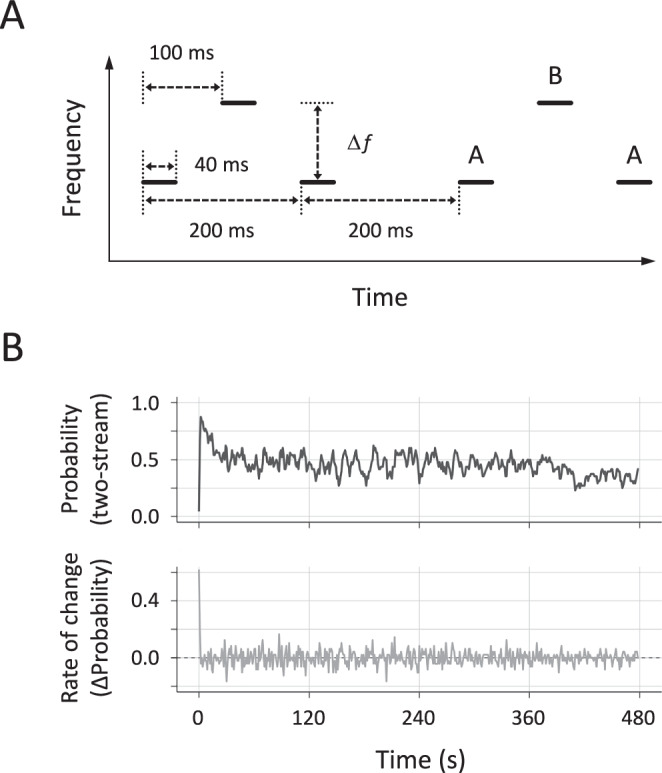
Stimulus design and group-averaged perceptual dynamics. **A** Schematic of the ABA-triplet sequence used in the auditory streaming task (total duration 480 s). Tones A (lower frequency) and B (higher frequency) define the sequence; Δ*f* denotes the A–B frequency separation. Participants reported perceptual switches between a one-stream percept (ABA-ABA-…) and a two-stream percept (A-A-A-A-… and -B---B--…). **B** Group-averaged time course (*N* = 48). Top panel: probability of reporting two streams at 0.1-s resolution (Gaussian-smoothed trace, *σ* = 0.3 s). Bottom panel: first discrete derivative (per second) of the smoothed probability.

Perception is an active process that relies not only on external sensory input but also on internal states and dispositional traits. A striking example is multistable perception, in which awareness stochastically switches between alternative interpretations of an unchanging stimulus^1–4^. In audition, the classic “streaming” paradigm has been extensively used to study the temporal dynamics of perceptual organization (Fig. 1A). In this paradigm, the stimulus consists of two pure tones A and B, at different frequencies, presented repeatedly in a triplet pattern ABA-ABA-ABA-… (with letters identifying tones and the hyphen denoting a silent gap). When listening to an ABA- sequence, listeners spontaneously alternate between an integrated “one-stream” percept, typically heard as a galloping pattern (ABA-ABA-…), and a segregated “two-stream” percept, typically heard as two concurrent isochronous streams (A-A-A-… and -B---B--…)^5–7^. Although methodologically simple, the paradigm captures a fundamental function of auditory scene analysis: parsing acoustic input into one or more perceptual objects that can, in principle, be mapped onto external causes. Such a function is essential for identifying sound sources, supporting communication in complex environments, and appreciating music^8^.

Interestingly, individual listeners show remarkably stable and idiosyncratic switching patterns in this task, which can persist over months or even years^9^. This suggests trait-like differences in auditory information processing even at the most basic level. For instance, higher switching rates, corresponding to more frequent exploration of alternative perceptual organizations, have been linked to ego-resiliency, a broad personality trait often described as greater flexibility in adapting to changing demands^10^. Here, we examined whether another trait, sensory processing sensitivity, was associated with the temporal dynamics of bistable auditory streaming. Sensory processing sensitivity, often measured by the Highly Sensitive Person Scale^11,12^, was originally introduced within personality psychology and has attracted growing interest in research on emotion, well-being, and atypical sensory–affective experiences^13–15^. Recent theoretical work has proposed that sensory processing sensitivity may be understood within a predictive-processing framework, in which individuals with greater sensitivity assign larger precision to incoming sensory signals^16^. Converging genetic and neuroimaging findings further support sensory processing sensitivity as a biologically anchored dimension related to environmental sensitivity^17^. Because auditory scene analysis depends on combining multiple acoustic cues over time^7^, sensory processing sensitivity may be relevant to how ambiguous sound sequences are parsed into coherent perceptual objects.

To test this hypothesis, we first used standard descriptive measures of the temporal dynamics of auditory streaming: percept durations, switching rates, and dominance-duration distributions^3,18,19^. However, we also aimed to move beyond phenomenological descriptions by using quantitative models. For auditory streaming, existing computational accounts include competition–adaptation dynamics and evidence‑accumulation formulations^20,21^. In neuromimetic models, for instance, simulated spiking activity in primary auditory cortex is fed into a saturating accumulation process^21^. Rather than fitting such neuromimetic models directly, we used an intermediate level of analysis between purely descriptive summaries and biologically specific modeling. Our goal was to estimate listener-level parameters with clear psychological interpretations by fitting interpretable models to the full dataset for each participant. This strategy allowed us to test whether trait-related differences primarily reflect a global bias toward one perceptual state or instead emerge in the maintenance of a percept once established. This distinction is theoretically motivated by recent predictive-processing accounts of bistable perception, which emphasize that perceptual experience depends on the dynamic balance between external sensory input and internally stabilizing influences over time^22^.

To briefly summarize the logic guiding the analyses that follow, the present study investigated whether sensory processing sensitivity was associated with the temporal dynamics of bistable auditory streaming, and which component of perceptual stability was most closely related to this trait. More specifically, we asked whether any trait-related association would be better explained by overall state occupancy or by state maintenance once a percept had been established. First, we quantified overall bias toward a given state as the proportion of time spent in that state and tested whether sensory processing sensitivity covaried with this state-occupancy measure. Second, we modeled time-resolved state maintenance using a discrete-time hazard approach, which estimates the momentary probability that an ongoing percept terminates, conditional on having lasted so far. Third, we adopted a first‑passage‑time perspective to link dominance‑duration distributions to candidate mechanisms for bistable perception, as this approach has been used in previous studies^23–25^. Finally, because these three frameworks approximate switching as a renewal (memoryless) process, we assessed short-range sequential dependence, mainly as a robustness check^26,27^.

## Methods

### Transparency and ethics statement

Prior to data collection, the target sample size (*N* = 46) was determined *a priori* using G*Power (ver. 3.1.9.7)^28^ for detecting bivariate correlations of *r* = 0.40 (*α* = 0.05 and power = 0.80). All analyses were conducted in R (ver. 4.5.1; https://www.r-project.org/). The study was not preregistered. The experimental protocol was approved by the Research Ethics Committee of Chukyo University (approval no. 2022–083) and was conducted in accordance with Ethical Guidelines for Medical and Biological Research Involving Human Subjects in Japan. Written informed consent was obtained from all participants prior to participation, and participants received JPY 1000 as compensation.

### Participants

Forty-eight participants (15 men and 33 women, based on self-reported gender; age range: 20–29 years, mean = 21.5, SD = 1.4) participated in the experiment. All participants reported normal hearing and no history of neurological or psychiatric illness, and all responded using their right hand. Information on socioeconomic status and race/ethnicity was not collected.

### Auditory streaming task

All participants completed the experiment individually in a quiet, sound-attenuated room. Auditory stimuli were delivered diotically through over-ear headphones (Sennheiser HD 599), with stimulus presentation and response collection controlled using Presentation software (Neurobehavioral Systems, Berkeley, CA, USA). The presentation level was set to 70 dB SPL, calibrated using an artificial ear (TYPE2015E, ACO, Tokyo). Before the main task, participants completed practice trials to become familiar with the stimulus structure and response method, and were instructed to report their perceptual experience continuously and precisely^10^. Response states were recorded with key-down timestamps at a sampling rate of 1000 Hz.

The experimental stimulus was a repeating ABA- sequence, in which A and B tones were pure tones at 891 Hz and 1122 Hz, respectively, thus separated by four semitones (Fig. 1A). Each tone lasted 40 ms with 10-ms cosine-squared onset and offset ramps. The stimulus onset asynchrony was fixed at 100 ms. The hyphen represents a silent gap. Stimulus parameters followed those used in previous studies^29–31^. Stimuli were generated in MATLAB (MathWorks, Natick, MA, USA) and rendered at 44.1 kHz sampling rate and 16-bit quantization. This stimulus configuration reliably elicits bistable auditory perception: participants typically alternate between an integrated one-stream percept (a galloping rhythm, ABA-ABA-…) and a segregated two-stream percept (two concurrent isochronous streams, A-A-A-A-… and -B---B--…).

Participants were instructed to continuously indicate their current percept by holding down one of two designated keys on a keyboard: one for the one-stream percept and the other for the two-stream percept. Key assignments were counterbalanced across participants. A key remained pressed as long as the percept persisted, and switching to the other key indicated a perceptual change. Each test trial lasted 8 min, during which the number and timing of perceptual switches were recorded. The task did not include a mixed/uncertain response option. Participants were instructed that, during brief ambiguous or transitional moments, they should keep holding the key corresponding to the preceding percept and switch only when the alternative percept became clearly dominant. They were also instructed to avoid rapid switching driven by momentary uncertainty.

### Assessment of sensory processing sensitivity

Individual differences in sensory processing sensitivity were measured with the Japanese version of the 27-item Highly Sensitive Person Scale^11^. The version used in this study was developed through rigorous back-translation procedures to ensure linguistic and conceptual equivalence^32,33^. Participants completed the questionnaire immediately after the auditory task. Items were rated on a 7-point Likert scale (1 = not at all, 7 = extremely). Example items include: “Are you bothered by intense stimuli, like loud noises or chaotic scenes?”, “Do you find it unpleasant to have a lot going on at once?”, and “Do you notice and enjoy delicate or fine scents, tastes, sounds, and works of art?” The questionnaire typically required about 10 min to complete. Internal consistency in the present sample was high (Cronbach’s *α* = 0.85). The Highly Sensitive Person Scale has been shown to demonstrate good discriminant validity from other major personality traits, such as neuroticism and extraversion^12^, supporting its specificity in assessing sensory–affective sensitivity. We conducted preliminary analyses on individual scores from the Highly Sensitive Person Scale. The scores, computed as the mean of 27 items, ranged from 3.22 to 6.15 (mean = 4.63, *SD* = 0.71). The distribution did not significantly deviate from normality (Shapiro–Wilk test, *W* = 0.98, *p* = 0.73). Correlations between perceptual measures and scores on the Highly Sensitive Person Scale are presented as descriptive summaries in the Results.

### Percept-duration distributions and preprocessing

Continuous responses were segmented into phases, defined as consecutive time intervals with an unchanged perceptual report (one-stream or two-stream). The duration of each phase was then used as the basic unit of analysis. To minimize artifacts from brief motor/reporting noise, durations shorter than 0.5 s were excluded. To reduce the influence of unusually long phases that may reflect lapses in continuous reporting, analyzed phase durations were truncated at 30 s; phases exceeding 30 s were excluded from model-based analyses. For each participant, we also computed (i) the total number of perceptual switches and (ii) the cumulative time spent in each perceptual state. The primary inferential analyses below used Bayesian models that directly targeted (i) overall state occupancy, (ii) time-resolved state termination (hazard), and (iii) distributional components of dominance durations. The occupancy model used Bayesian beta regression, the hazard model used a Bernoulli mixed-effects specification, and the Gamma model used distributional regression.

### Priors and posterior inference

In Bayesian models, priors encoded reasonable ranges for parameters before the data were observed. We used weakly informative priors to regularize estimation, especially in later time bins where data are sparse. For the occupancy Beta regression, *β*_0_ and *β*_1_ followed Normal(0, 0.5) priors and the precision parameter *ϕ* followed Exponential(1). For the discrete-time hazard model (Bernoulli with a complementary log–log (cloglog) link), we specified a Student-*t*(3, −7, 2) prior on the intercept, which concentrates prior mass on very low early per-bin termination probabilities on the probability scale. For the Gamma distributional regression, priors were Normal(0, 0.5) for coefficients, Student-*t*(3, 0, 1) for intercepts, and Exponential(1) for the random-intercept SD.

Posterior sampling was performed using Hamiltonian Monte Carlo with the No-U-Turn Sampler (NUTS) in Stan (via brms), using 4 chains with 4000 iterations per chain (2000 warmup). We set a target acceptance rate of 0.95 and a maximum tree depth of 12 to improve sampling stability. Convergence and sampling quality were assessed using *R̂*, effective sample sizes, and inspection of sampler diagnostics (e.g., divergent transitions). Posterior uncertainty is summarized using posterior means and 95% credible intervals (CrIs).

### State occupancy

To quantify overall perceptual bias, we computed for each participant *j* the proportion of total reporting time spent in the two-stream state:

1 $${{{{\rm{occ}}}}}_{{two},j}=\frac{{T}_{{two},\,j}}{{T}_{{one},j}+{T}_{{two},j}},$$

where *T*_*one,j*_ and *T*_*two,j*_ denote the summed dominance durations in one-stream and two-stream states, respectively. Because the two occupancies are complementary, this measure fully summarizes overall state bias. We modeled the observed occupancy occ_*two,j*_ as a beta-distributed outcome with mean *u*_*j*_. To account for the bounded nature of the data while allowing the variance to depend on the mean, we used a Bayesian beta regression with a logit link. Because beta regression requires responses to lie strictly within the open interval (0, 1), any exact 0 or 1 occupancy values were adjusted slightly inward by a negligible amount before model fitting. We modeled the expected occupancy *u*_*j*_ as

2 $${{{\rm{logit}}}}\left({u}_{j}\right)={\beta }_{0}+{\beta }_{{{{\rm{hsp}}}}}{{{{\rm{hsp}}}}}_{z,j}.$$

Here, hsp_*z,j*_ denotes the participant-level standardized score on the Highly Sensitive Person Scale.

### Discrete-time hazard model of state termination

To characterize within-phase stabilization dynamics, each dominance phase was partitioned into consecutive 100-ms bins (Δ*t* = 0.1 s). This representation converted each continuous dominance phase into a sequence of discrete time intervals, yielding a person-period dataset for discrete-time survival analysis. We used the following notation throughout the modeling sections: participant *j* = 1, …, *J*; dominance phase *i* = 1, …, *N*_*j*_ within participant *j*; and time bin *k* = 1, …, *n*_*ij*_, within phase *i*. The perceptual state is coded as state_*ij*_ = 0 for one-stream and state_*ij*_ = 1 for two-stream. For phase *i* of participant *j* within duration *d*_*ij*_, we define ${n}_{ij}=\lceil {d}_{ij}/\varDelta t\rceil$ bins indexed by *k* = 1, …, *n*_*ij*_, with bin mid-times *t*_*ijk*_ = (*k* – 0.5)Δ*t*. A binary termination indicator *y*_*ijk*_ is set to 1 for the final bin (*k* = *n*_*ij*_) and 0 otherwise. This corresponds to a discrete-time hazard

3 $$h\left({t}_{{ijk}}\right)=\Pr \left({y}_{{ijk}}=1|{y}_{{ij}1}=\cdot \cdot \cdot ={y}_{{ij},k-1}=0\right),$$

i.e., the probability that a phase terminates within the current 100-ms bin given that it has persisted up to that bin.

We modeled *y*_*ijk*_ using a Bayesian Bernoulli mixed-effects model with a cloglog link, which connects naturally to a continuous-time hazard interpretation under fine binning:

4 $${{{\rm{cloglog}}}}\left\{h\left({t}_{{ijk}}\right)\right\}= 	{\beta }_{0}+f\left({t}_{{ijk}}\right)+{\beta }_{{{{\rm{state}}}}}{{{{\rm{state}}}}}_{{ij}}+{f}_{{{{\rm{state}}}}}\left({t}_{{ijk}}\right){{{{\rm{state}}}}}_{{ij}}\\ 	+{\beta }_{{{{\rm{hsp}}}}}{{{{\rm{hsp}}}}}_{z,j}+{\beta }_{{{\mathrm{int}}}}\left({{{{\rm{state}}}}}_{{ij}}\times {{{{\rm{hsp}}}}}_{z,j}\right)+{b}_{j},{b}_{j}{{{\mathscr{ \sim }}}}{{{\mathscr{N}}}}\left(0,\,{\sigma }_{b}^{2}\right),$$

where *f*(*t*) is a natural cubic spline of elapsed time implemented as ns(*t*, *df* = 4), and *b*_*j*_ is a participant-specific random intercept. The spline term appears twice because the model includes both (i) the overall time course of the hazard and (ii) a state-by-time interaction, allowing that time course to differ between the one-stream and two-stream states. The interaction (*β*_int_) further allows effects of sensory processing sensitivity to differ between states. Planned posterior contrasts were computed at prespecified time points (Supplementary Table 1). To document how much data contributed to later time bins, we additionally derived curves showing the proportion of phases still ongoing over time (Supplementary Fig. 1).

### Gamma distributional regression of dominance durations

To characterize dominance-duration distributions in an interpretable way, we used a Bayesian Gamma distributional regression that decomposed durations into (i) an overall timescale (mean, *μ*) and (ii) a shape/regularity component (*κ*). Let *y*_*ij*_ > 0 denote the *i*-th dominance duration from participant *j*, and let state_*ij*_ indicate the perceptual state (one-stream vs. two-stream). Sensory processing sensitivity was entered as hsp_*z,j*_. We used the mean–shape parameterization of the Gamma distribution, where

5 $$\begin{array}{cc}E\left[{y}_{{ij}}\right]={\mu }_{{ij}}, & {{{\rm{Var}}}}\left({y}_{{ij}}\right)={\mu }_{{ij}}^{2}/{\kappa }_{{ij}}.\end{array}$$

Under this parameterization, *μ*_*ij*_ sets the overall timescale, whereas *κ*_*ij*_ controls relative variability (the coefficient of variation equals 1/√*κ*_*ij*_). Both components were modeled with log links to ensure positivity.

For the model of the mean component, we included perceptual state, sensory processing sensitivity, their interaction, and a participant-specific random intercept to capture stable between-participant differences in overall dominance timescales:

6 $$\log {\mu }_{{ij}}={\beta }_{0}+{\beta }_{{{{\rm{state}}}}}{{{{\rm{state}}}}}_{{ij}}+{\beta }_{{{{\rm{hsp}}}}}{{{{\rm{hsp}}}}}_{z,j}+{\beta }_{{{\mathrm{int}}}}\left({{{{\rm{state}}}}}_{{ij}}\times {{{{\rm{hsp}}}}}_{z,j}\right)+{b}_{j},{b}_{j}{{{\mathscr{ \sim }}}}{{{\mathscr{N}}}}\left(0,\,{\sigma }_{b}^{2}\right).$$

For the model of the shape component, we modeled temporal regularity as

7 $$\log {\kappa }_{{ij}}={\alpha }_{0}+{\alpha }_{{{{\rm{state}}}}}{{{{\rm{state}}}}}_{{ij}}+{\alpha }_{{{{\rm{hsp}}}}}{{{{\rm{hsp}}}}}_{z,j}+{\alpha }_{{{\mathrm{int}}}}\left({{{{\rm{state}}}}}_{{ij}}\times {{{{\rm{hsp}}}}}_{z,j}\right).$$

Participant-level random intercepts were included for the mean component, because between-participant differences in overall dominance timescale were a primary focus of the analysis. The shape component was modeled with fixed effects only, as participant-level random effects on shape would substantially increase model complexity and are typically difficult to estimate stably from phase-duration data.

### Sequential dependence as a robustness check

Because phase-based analyses often approximate switching as a renewal process, we evaluated short-range sequential dependence as a robustness check using within-state autocorrelations and parametric kernel fits. Full details are reported in Supplementary Methods.

## Results

### Phenomenological measures of auditory streaming

We first analyzed the probability of each perceptual state over time. As shown in Fig. 1B, at the group level, the probability of reporting two streams rapidly stabilized and remained near 50% with modest fluctuations throughout the 480-s sequence (0.1-s resolution; Gaussian smoothing, *σ* = 0.3 s). Because smoothing can obscure slow trends, we additionally quantified time-dependent drift using unsmoothed reports aggregated into short time windows (see Supplementary Note 1). These analyses showed a small downward drift on the probability scale, corresponding to a decrease of a few percentage points per minute. We therefore treated the sequence as eliciting a broadly balanced, and thus sensitive, bistable regime at the group level.

Percept duration distributions were right‑skewed (see the section on Gamma first-passage decomposition), as is expected in a bistable streaming paradigm^3^. We summarized the central tendency with the median as a robust statistic. Median durations were comparable across states: one-stream, 6.04 s (IQR: 3.24–10.36 s); two-stream, 6.12 s (IQR: 3.41–11.23 s), further indicating that stimulus parameters produced a balanced bistable regime.

**Fig. 2:**
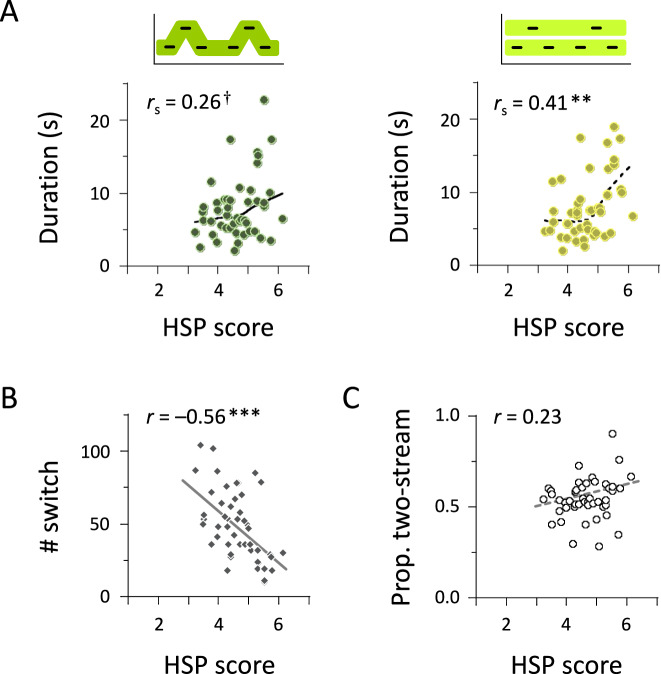
Relationships between sensory processing sensitivity and perceptual dynamics during auditory streaming. **A** Associations between scores on the Highly Sensitive Person (HSP) Scale and median percept durations for one-stream (left) and two-stream (right) states. Points indicate individual participants (*N* = 48). Medians are used as robust summaries for right‑skewed durations. Spearman’s rank correlation coefficients (*r*_s_) are reported; trend lines were estimated using LOWESS (locally weighted scatterplot smoothing) to illustrate monotonic, potentially non-linear relations. **B** Negative correlation between HSP score and the number of perceptual switches across 8-min sequences. Pearson’s correlation (*r*) is reported. **C** Relation between HSP score and the proportion of time spent in the two-stream state. ***p < 0.001, **p < 0.01, ^†^*p* < 0.10 (two-tailed).

Next, we examined whether individual differences in sensory processing sensitivity, indexed quantitatively by scores on the Highly Sensitive Person Scale, were correlated with descriptive measures of bistable auditory streaming (Fig. 2). Higher scores were associated with longer percept durations for the two-stream state (*r*_s_ = 0.41, *p* = 0.004, 95% confidence interval (CI) [0.13, 0.64]; ~17% of the total variance; Fig. 2A). The corresponding association for the one-stream state was smaller and statistically less certain (*r*_s_ = 0.26, *p* = 0.077, 95% CI [–0.04, 0.52]). The strongest association was found with the total number of perceptual switches (Fig. 2B), with a negative correlation demonstrating fewer switches for participants with higher scores (*r* = –0.56, *p* < 0.001, 95% CI [–0.74, –0.32]; ~31% of the total variance). Finally, the proportion of time spent in the two-stream state did not show any significant correlation with scores on the Highly Sensitive Person Scale (*r* = 0.23, *p* = 0.12, 95% CI [–0.08, 0.49]; Fig. 2C).

To summarize, higher scores on the Highly Sensitive Person Scale were found to correlate with longer percept durations in the two-stream state and fewer switches. Thus, greater sensory processing sensitivity was associated with greater perceptual stability. However, phenomenological stability can reflect at least two conceptually separable mechanisms: an overall bias toward occupying a given perceptual state (state occupancy) and/or a reduced likelihood of terminating that state once it is established (state maintenance). To distinguish these components, we turned to modeling.

### State occupancy and time-resolved percept stabilization

We first addressed the possibility of an overall bias toward segregation. For each participant, we modeled the proportion of total time spent in the segregated two-stream state using a Bayesian beta regression with a logit link (*N* = 48). The posterior distribution showed little evidence that greater sensory processing sensitivity shifted the overall balance toward the two-stream percept (*β*_hsp_ = −0.04, 95% CrI [−0.16, 0.07]). On an odds scale, the posterior mean corresponds to an odds ratio of exp(*β*_hsp_) ≈ 0.96 for a 1-SD increase in Highly Sensitive Person Scale scores, with substantial posterior mass spanning no effect.

**Fig. 3:**
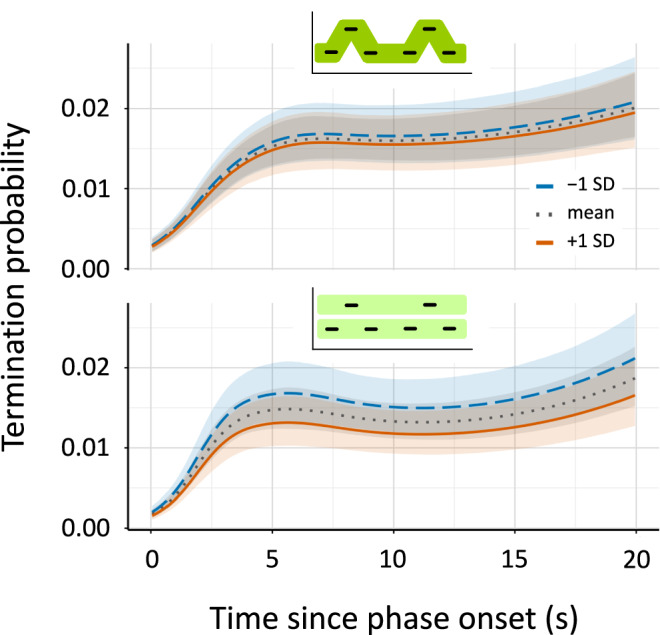
Time-resolved termination probability as a function of sensory processing sensitivity. Per-bin termination probability (100-ms bins) is plotted as a function of time since the onset of dominance phases, separately for the integrated one-stream percept (top) and the segregated two-stream percept (bottom). Curves show posterior estimates from the Bayesian discrete-time hazard model (cloglog link) evaluated at three levels of sensory processing sensitivity, namely, scores on the Highly Sensitive Person Scale: −1 SD, mean, and +1 SD (standardized within sample). Shaded bands indicate 95% credible intervals.

We next examined the time course of percept maintenance. Specifically, we used a discrete-time hazard model to estimate the momentary probability that an ongoing percept terminated (Fig. 3). We fit a Bayesian Bernoulli mixed-effects model (with a cloglog link) to a 100-ms person-period dataset, with a natural-spline time term (*df* = 4), perceptual state, sensory processing sensitivity, and their interaction, along with participant-specific random intercepts. The model captured strong time dependence in termination probability and substantial individual differences in baseline termination tendency (random-intercept SD = 0.47, 95% CrI [0.37, 0.59] on the linear predictor scale). The main effect of sensory processing sensitivity on termination within the one-stream state was small and uncertain (*β*_hsp_ = −0.03, 95% CrI [−0.19, 0.11]). However, sensory processing sensitivity showed a negative association with termination in the two-stream state, as reflected in the interaction term (*β*_int_ = −0.09, 95% CrI [−0.18, 0.00]). This pattern is consistent with the interpretation that greater sensory processing sensitivity was associated with enhanced maintenance of the two-stream state once established.

To further characterize the time course of this stabilization process, we summarized posterior contrasts in termination probability between the two- and one-stream states (Δhaz(*t*) = haz_two_(*t*) − haz_one_(*t*)) at prespecified time points and three levels of sensory processing sensitivity (hsp_z_ = −1, 0, +1; Fig. 3). For average and greater sensory processing sensitivity, Δhaz(*t*) became credibly negative in the later portion of the dominance phase, indicating lower termination probability, i.e., greater stabilization, for the two-stream percept relative to the one-stream percept (Supplementary Table 1). For example, at 12 s after phase onset, the posterior mean difference was −0.00292 (for hsp_z_ = 0: 95% CrI [−0.00534, −0.00064], *P*(Δhaz < 0) = 0.994) and −0.00395 (for hsp_z_ = +1: 95% CrI [−0.00667, −0.00138], *P*(Δhaz < 0) = 0.999). A similar pattern was observed at 8 s (hsp_z_ = 0: −0.00217, 95% CrI [−0.00410, −0.00031], *P*(Δhaz < 0) = 0.989; hsp_z_ = +1: −0.00328, 95% CrI [−0.00572, −0.00097], *P*(Δhaz < 0) = 0.997) and at 16 s (hsp_z_ = +1: −0.00387, 95% CrI [−0.00711, −0.00079], *P*(Δhaz < 0) = 0.994). Because estimates at longer elapsed times were supported by progressively fewer phases, these late-bin divergences should be interpreted cautiously. For transparency, the proportion of phases remaining over time is shown as separate survival curves in Supplementary Fig. 1.

To summarize, the occupancy and hazard models together provided little evidence that sensory processing sensitivity was associated with a global bias toward the two-stream state. By contrast, they did suggest that greater sensory processing sensitivity was more closely linked to enhanced maintenance of the two-stream percept once established.

### Gamma first‑passage decomposition of timescale and regularity

**Fig. 4:**
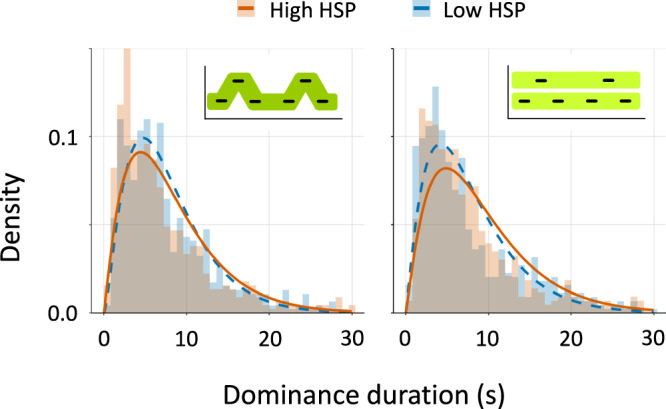
Dominance-duration distributions and fitted Gamma densities by perceptual state and Highly Sensitive Person (HSP) group. Histograms show the empirical distribution of dominance durations for the one-stream (left) and two-stream (right) percepts, plotted separately for high and low HSP groups (median split; *n* = 24 for each). Overlaid curves show the corresponding fitted Gamma densities derived from the Bayesian Gamma regression, illustrating how the distributional shape varies across perceptual states and individual differences in sensory processing sensitivity.

We next characterized the full distribution of dominance durations using a Bayesian Gamma distributional regression (*N* = 2235 phases, 48 participants; Fig. 4). Perceptual switching can be described in a broad first‑passage‑time framework: a percept ends when an underlying stochastic process reaches a switching criterion. We used the Gamma/Erlang family of distributions as a descriptive model because it decomposes the duration distribution into two easily interpretable components: the mean (*μ*), which sets the overall timescale of the underlying process, and the shape (*κ*), which indexes its temporal regularity. The distribution variance is *μ*^2^/*κ*, so larger *κ* corresponds to a tighter distribution of durations around the mean, whereas smaller *κ* implies more variable durations and a more strongly long-tailed distribution.

At the average level of sensory processing sensitivity (hsp_z_ = 0) in the one-stream state, the estimated mean duration was *μ* ≈ 8.00 s (exp(*β*_0_), 95% CrI [7.10, 9.03]) and the estimated shape was *κ* ≈ 2.34 (exp(*α*_0_), 95% CrI [2.16, 2.53]). Mean durations were only slightly longer in the two-stream state (*β*_state_ = 0.06, 95% CrI [0.00, 0.12]), and sensory processing sensitivity showed little evidence for a main effect on the mean duration within the one-stream state (*β*_hsp_ = 0.03, 95% CrI [−0.09, 0.14]). Critically, the association between sensory processing sensitivity and mean duration was stronger in the two-stream state, as indicated by a positive interaction in the mean component (*β*_int_ = 0.06, 95% CrI [0.00, 0.12]). On the natural scale, each 1-SD increase in Highly Sensitive Person Scale scores was associated with a 6.7% greater mean duration for two-stream than for one-stream phases (exp(*β*_int_) = 1.067, 95% CrI [1.004, 1.131], posterior probability of an increase = 0.981). This interaction therefore indicates that greater sensory processing sensitivity was more strongly associated with longer two-stream phases, although it does not by itself exclude weaker effects in the one-stream state.

In the shape component, higher scores were associated with a modest reduction in *κ* (*α*_hsp_ = −0.10, 95% CrI [−0.18, −0.01]; exp(*α*_hsp_) = 0.910, 95% CrI [0.837, 0.986], posterior probability of a decrease = 0.987), whereas evidence for a state-specific modulation of *κ* was weak (*α*_int_ = 0.10, 95% CrI [−0.02, 0.21]).

Dominance durations also varied substantially across participants in their overall timescale, which we accommodated by including a participant-specific random intercept in the mean model. The estimated variability of this random intercept was SD = 0.36 on the log-mean scale (95% CrI [0.29, 0.46]), corresponding to approximately exp(0.36) ≈ 1.43-fold differences in baseline timescales across participants. The Bayesian framework made it possible to estimate these between-participant differences directly while propagating their uncertainty into the fixed-effect estimates.

### Short-range history effects

As all analyses reported so far treated successive phases as approximately independent, we directly evaluated this modeling assumption. To do so, we quantified sequential dependence in log-transformed durations within each perceptual state, using autocorrelation as a measure of short-range history dependence. Autocorrelations were positive at short lags and declined with increasing lag, indicating a brief carry-over effect that disappeared within a few returns of the same percept. Details of the lag-wise autocorrelations, parametric kernel fits, and model comparisons are reported in Supplementary Note 2 and Supplementary Fig. 2. Thus, the assumption of strict independence was not fully met over short time scales. Crucially, however, there was no evidence that sensory processing sensitivity modulated these sequential effects. Accordingly, the main inferences drawn from the occupancy, hazard, and Gamma models are unlikely to be explained by short-range history dependence.

## Discussion

We found that sensory processing sensitivity was associated with systematic differences in the temporal dynamics of auditory scene analysis. At the phenomenological level, greater sensory processing sensitivity was associated with fewer perceptual switches overall, indicating greater perceptual stability. Differences in Highly Sensitive Person Scale scores accounted for 32% of the between-participant variance in the total number of switches. A corresponding association with percept duration was observed most clearly for the two-stream percept, for which sensory processing sensitivity accounted for ~17% of the between-participant variance. Taken together, these descriptive findings indicate that sensory processing sensitivity was meaningfully related to the stability of auditory scene analysis at the phenomenological level.

Using interpretable models, we then asked which aspect of perceptual stability was most closely related to sensory processing sensitivity. The occupancy analysis provided little evidence for a global bias toward either perceptual state, and the hazard analysis likewise provided little evidence for a generic slowing of switching dynamics across both states. Instead, converging time-resolved and distributional analyses pointed to a relatively stronger association with the maintenance of the two-stream percept once established. In the discrete-time hazard model, sensory processing sensitivity showed a more negative association with termination probability in the two-stream state than in the one-stream state. In the Gamma/Erlang model, sensory processing sensitivity was associated with longer dominance timescales, particularly for two-stream phases. We therefore summarize our findings as suggesting that sensory processing sensitivity stabilized the segregated two-stream percept once established.

### Predictive processing in auditory streaming and sensory processing sensitivity

Our modeling approach targets an intermediate level of explanation, between purely phenomenological descriptions of behavior and fully specified neural models. At this level, predictive-processing accounts provide a useful conceptual bridge for interpreting the present findings. Such accounts propose that perceptual inference combines ambiguous sensory input with internally generated expectations to estimate the most plausible external cause of the current sensory state^34,35^. This general framework is particularly relevant to bistable perception, where the sensory input underdetermines a single, obvious interpretation. Recent computational work has further suggested that perceptual switches may occur when the currently favored interpretation no longer provides an adequate account of the incoming input^36^.

For auditory streaming, predictive accounts have also been proposed in several previous studies^19,37,38^. In these models, candidate perceptual organizations compete with one another, and their relative stability depends in part on how well each organization predicts the temporal structure of the sound sequence. This framework has received experimental support from studies manipulating acoustic regularity, which showed that increasing regularity selectively stabilized the segregated two-stream percept without strengthening the integrated one-stream percept^39^. This pattern is similar to the one observed here, where sensory processing sensitivity was linked more strongly to the maintenance of the two-stream percept than to a global shift in perceptual occupancy.

A further reason to consider this framework comes from recent theorizing about sensory processing sensitivity itself. It has been proposed that sensory processing sensitivity may be understood within a predictive-processing perspective, in which highly sensitive persons assign relatively greater precision to incoming sensory signals^16^. This account would predict that subtle but reliable acoustic structure carries greater weight in perceptual inference for such listeners. In the present context, one possible interpretation is that greater sensory processing sensitivity increased the influence of temporal regularities supporting segregation, thereby favoring maintenance of the segregated two-stream percept. This interpretation remains speculative, but it offers a coherent way to link our findings with both predictive accounts of auditory streaming and broader theories of sensory processing sensitivity.

Recent work also suggests that bistable perception may depend on slow fluctuations in the relative balance between externally driven and internally stabilizing modes of inference, rather than on a fixed weighting of sensory evidence and prior expectations^22^. The present results are compatible with the broader idea that sensory processing sensitivity was related less to a global perceptual bias than to the temporal stabilization of an already established percept. What the present data do suggest is that sensory processing sensitivity was linked to how perceptual organization evolved over time, and not simply to which percept dominated overall.

### Broader implications and candidate substrates

Our results suggest that trait-linked modulation of perceptual organization is not a unitary phenomenon. By separating overall state occupancy, time-resolved termination dynamics, and distribution-level properties of dominance durations, we identified conceptually distinct components of perceptual stability. This decomposition may prove useful for interpreting other individual differences previously reported in auditory multistability. For example, switching slows with age in auditory streaming^29^, but it remains unclear whether this reflects altered state occupancy, within-phase termination dynamics, or a global shift in dominance timescale. A similar logic may also help clarify trait-related differences in verbal transformations, including those linked to autism- and schizophrenia-spectrum traits^31,40–42^. Although verbal transformations depend partly on processes distinct from those involved in auditory streaming^31,43^, the same analytic framework may help distinguish shared from task-specific mechanisms across auditory multistable paradigms.

Established neural correlates of auditory streaming provide a further context for the present findings. Auditory streaming recruits distributed circuits along peripheral and central auditory pathways^44–48^, with additional contributions from parietal and cerebellar regions^35,49^. Specifically, event-related functional neuroimaging demonstrated that switch initiation alternates between the auditory thalamus and auditory cortex, depending on whether the switch moves toward or away from the currently dominant percept^47^. Magnetic resonance spectroscopy has further shown that higher ratios of gamma-aminobutyric acid (GABA) to glutamate–glutamine (Glx) in the auditory cortex are correlated with longer percept durations^30^. Tentatively, stabilization associated with sensory processing sensitivity may be implemented through neural processes involved in maintaining perceptual states, e.g., local competition and adaptation within thalamo–cortical circuits. Future studies that combine streaming with measures of network-level engagement or excitation–inhibition balance could test whether the trait-linked stabilization observed here is accompanied by corresponding signatures in auditory cortical and thalamo–cortical processing.

Finally, we can situate our findings within the broader literature on sensory processing sensitivity. Higher scores on the Highly Sensitive Person Scale were associated with enhanced maintenance of the two-stream percept once established. This pattern is consistent with a listening style that places relatively greater weight on fine-grained acoustic structure, a tendency that may be beneficial for source separation in complex scenes such as speech-in-noise or polyphonic music. At the same time, emphasizing local detail could come at a cost when perceptual integration is advantageous, potentially increasing the risk of over-segregation or reduced global coherence in highly variable environments. Such context-dependent trade-offs complement the argument that sensory processing sensitivity entails both benefits and costs, with the balance shaped by environmental structure^11,12,14,16,17^. This yields a testable prediction: stable, reliable segregation cues should disproportionately benefit listeners with greater sensory processing sensitivity, whereas conflicting or rapidly changing cues may more readily trigger over-segregation.

### Limitations

First, our finding that sensory processing sensitivity was clearly linked to the two-stream state, without affecting the overall balance between states, may appear paradoxical. One possibility is that sensory processing sensitivity influenced both perceptual states, but that the corresponding effect in the one-stream state remained too small to be detected statistically in the present dataset. We acknowledge this as a methodological possibility. At the same time, the Bayesian models yielded interactions between sensory processing sensitivity and perceptual state. This indicates that the case for state dependence does not rest solely on the absence of a clear effect in the one-stream state.

Second, our modeling approach assumed that switches could be approximated as conditionally independent events in a renewal-like process^4^. To evaluate this simplifying assumption, we further examined short-range sequential dependence in dominance durations. Reports of such dependencies have been mixed in previous studies, in both audition and vision^3,26,27,50^. In our data, sequential dependence was present but short-lived. This is consistent with a rapidly decaying carry-over component that disappeared within a few returns of the same percept. Importantly, this carry-over component showed little evidence of being moderated by sensory processing sensitivity, making it unlikely that the main model-based inferences were driven by short-range history effects. Should this assumption fail more substantially in other paradigms, alternative modeling frameworks may be required.

Third, we looked for perceptual mechanisms mediating the influence of sensory processing sensitivity. However, non-perceptual response-related factors could also have contributed. In particular, in our continuous-reporting paradigm, personality traits related to sensory processing sensitivity may have influenced behavior through sustained attention over long durations, deliberately cautious response strategies, or compliance with task instructions^16^. Future studies could incorporate additional measures—such as reports of uncertain perceptual states, response latencies, catch trials, or attentional diversion manipulations—to better constrain such possibilities. Even so, the fact that the present effects were state-dependent makes a purely non-perceptual explanation less straightforward.

Other methodological limitations should also be noted. Our sample consisted of healthy young adults, limiting generalizability to other age groups^29,51^ and to clinical populations^40^. We focused on neutral listening in a streaming paradigm with fixed stimulus parameters (e.g., Δ*f* and stimulus onset asynchrony); therefore, the extent to which our findings generalize to conditions involving volitional control^3,30^, to other forms of perceptual ambiguity^52,53^, or to broader stimulus regimes^54^ remains to be established. Finally, sensory processing sensitivity was measured by self-report using the Highly Sensitive Person Scale^11^. Future studies could strengthen individual estimates of this trait by combining self-report measures with behavioral and neural indices of sensory processing.

## Conclusion

Sensory processing sensitivity was associated with systematic differences in the temporal dynamics of auditory perceptual organization. Participants with higher scores on the Highly Sensitive Person Scale showed fewer switches overall and longer percept durations in the segregated two-stream state. This pattern was most consistent with enhanced maintenance of the two-stream state once established, rather than a global shift in overall state occupancy. In the context of rhythmic cognition, our findings highlight how endogenous personality traits interact with exogenous temporal structure to shape perceptual organization over time.

## Supplementary information

Transparent Peer Review file

Supplementary Information

## Data Availability

The dataset used in this study is available in the Open Science Framework (OSF) repository at 10.17605/OSF.IO/T6MGK.

## Code availability

The analysis code used in this study is available on OSF at 10.17605/OSF.IO/T6MGK.
