# Supplementary material for: Sensory processing sensitivity is associated with state-dependent stabilization of perceptual organization in auditory streaming
Source: Commun Psychol. 2026 Jun 9;4:99. doi: 10.1038/s44271-026-00482-z (PMC13319501; doi:10.1038/s44271-026-00482-z)
Supplement: Supplementary file 2 — Supplementary Information [file 44271_2026_482_MOESM2_ESM.pdf]

Supplementary Information

# Sensory processing sensitivity is associated with state-dependent stabilization of perceptual organization in auditory streaming

Hirohito M. Kondo[1,*] and Daniel Pressnitzer[2]

[1]School of Psychology, Chukyo University, Nagoya, Aichi 466-8666, Japan

[2]Laboratoire des systèmes perceptifs, Département d'études cognitives, École normale supérieure, PSL University, CNRS, 75005 Paris, France

***Corresponding author**

Hirohito M. Kondo, Ph.D. https://orcid.org/0000-0002-7444-4996

School of Psychology

Chukyo University

101-2 Yagoto Honmachi, Showa, Nagoya, Aichi 466-8666, Japan

E-mail: kondo@lets.chukyo-u.ac.jp

# Supplementary Methods

## Short-range sequential dependence and kernel-based characterization

We examined whether the duration of a given perceptual state was statistically related to durations of subsequent recurrences of the same state. This analysis allowed us to test whether perceptual switching could be approximated as memoryless processes or instead showed short-range temporal dependencies. Classical accounts often approximate bistable perception as a renewal process with an exponential kernel, implying independence between successive dominance durations [1]. By contrast, deviations from exponentiality—such as power-law scaling of history dependence across percept durations—would suggest a heavier-tailed process with long-range memory components [2].

To assess whether dominance durations showed sequential dependencies, we first computed the autocorrelation function across successive percepts. For each participant and perceptual state, we obtained correlations between the current log-transformed duration $y_t$ and its lagged values $y_{t-g}$ for lags $g = 1, ..., G$.

These calculations were implemented in R (ver. 4.5.1; https://www.r-project.org/) using the acf() function. Formally, the lag-$g$ autocorrelation coefficient ($\rho$) was defined as

$$\rho_g = \frac{\mathrm{Cov}(y_t, y_{t-g})}{\sqrt{\mathrm{Var}(y_t)\,\mathrm{Var}(y_{t-g})}}. \tag{1}$$

Group-level summaries were obtained by averaging these coefficients across participants, and inferential statistics are reported below.

## Kernel-based modeling of history effects

To formalize the decay structure of sequential dependencies, we modeled the expected influence of past percepts using parametric kernels. Specifically, we considered two competing formulations.

Exponential kernel (memoryless decay):

$$w(g; \lambda) = \lambda^{g-1},\ 0 < \lambda < 1, \tag{2}$$

which assumes that the contribution of a percept decays exponentially with lag.

Power-law kernel (scale-free decay):

$$w(g;\alpha) = g^{-\alpha},\ \alpha > 0, \tag{3}$$

which assumes that contributions diminish according to a power law, preserving long-tailed influences. In both cases, the kernel weights were normalized with respect to $w(1) = 1$ for comparability.

For each kernel, the predicted weighted history term was computed as

$$W_t = \sum_{g=1}^{G} w(g;\theta)\, y_{t-g} \tag{4}$$

where $\theta \in \{\lambda, \alpha\}$ denotes the kernel parameter. The current duration was then modeled using a linear mixed-effects model,

$$y_t = \beta_0 + \beta_1 W_t + b_j,\ b_j \sim \mathcal{N}(0, \sigma_b^2), \tag{5}$$

with additional covariates, i.e., percept type and standardized score on the Highly Sensitive Person Scale, included in extended models. Parameters were estimated via restricted maximum likelihood. Model fit was evaluated using Akaike information criterion (AIC).

To provide an interpretable measure of memory span, we computed the equivalent half-life *h* of each kernel, defined as the lag at which the kernel weight decays to half its initial value.

Exponential kernel:

$$w(h;\lambda) = 0.5\ \Rightarrow\ h_{\exp} = 1 + \frac{\ln 0.5}{\ln \lambda} \tag{6}$$

Power-law kernel:

$$h^{-\alpha} = 0.5\ \Rightarrow\ h_{\text{pow}} = 2^{1/\alpha}. \tag{7}$$

Multiplying these phase-based values by the median percept duration (in seconds) yielded half-life estimates on the physical timescale. This transformation allowed us to visualize kernel decay not only in terms of percepts but also in absolute time, highlighting the difference in memory span over which prior percepts continue to modulate.

## Supplementary Note 1

### Slow drift in two-stream reporting over the trial

To assess whether there was a slow drift in perceptual reporting across the 480-s sequence, we checked time trends in two-stream reports using a windowed mixed-effects analysis. We first aggregated unsmoothed binary reports into overlapping time windows (20-s width, 10-s step; excluding the first 10 s). For each participant and window, we computed the number of two-stream samples and total samples. We then fit a binomial generalized linear mixed model with a logit link, including a fixed effect of elapsed time (centered and expressed in minutes) and participant-specific random intercepts and slopes. This analysis revealed a small but reliable negative time trend, indicating slightly reduced two-stream reporting later in the trial (fixed effect of time: $\beta = -0.143$, SE = 0.048, $z = -2.97$, $p = 0.003$). On the probability scale, this corresponds to an approximate decrease of 3–4 percentage points per minute around the mid-range of the response distribution: a rough conversion from the logit slope, evaluated near $p \approx 0.5$. Together, these results suggest that any slow drift was modest in magnitude and does not alter the interpretation that the stimulus parameters supported a broadly balanced bistable regime at the group level.

## Supplementary Note 2

### Sequential dependence in dominance durations

We quantified sequential dependence using the autocorrelation ($\rho_g$) of log-transformed durations within each perceptual state, following previous studies on bistable perception [3,4]. By construction, lag-1 ($\rho_1$) compares a percept to the next recurrence of the same percept, separated by one intervening phase of the other percept, i.e., two switches apart. Group-level analyses revealed short-range, positive dependencies (**Supplementary Figure 2A**). For the one-stream percept, $\rho_1$ was greater than zero ($\rho_1 = 0.113$; Wilcoxon signed-rank test, $p = 0.002$), whereas subsequent lags were weaker ($\rho_2 = 0.048$, $p = 0.065$; $\rho_3 = 0.038$, $p = 0.18$; $\rho_4 = 0.035$, $p = 0.085$; $\rho_5 = -0.037$, $p = 0.16$). For the two-stream percept, significant positive autocorrelations extended to lag-3 ($\rho_1 = 0.111$, $p = 0.006$; $\rho_2 = 0.100$, $p = 0.002$; $\rho_3 = 0.082$, $p = 0.006$) and dissipated thereafter ($\rho_4 = 0.00$, $p = 0.99$; $\rho_5 = -0.021$,

$p$ = 0.66). A linear mixed-effects model confirmed a monotonic decline of autocorrelation with lag ($\beta_{lag}$ = –0.031, 95% Wald confidence interval (CI) [–0.045, –0.023]; $\chi^2_{(1)}$ = 3.84, $p$ = 0.050), with no evidence for a lag × percept interaction ($\beta_{lag \times percept}$ = 0.028, 95% Wald CI [–0.020, 0.044]; $\chi^2_{(1)}$ = 0.21, $p$ = 0.65). Thus, positive $\rho$ values at short lags indicate a carry-over effect: longer percept durations tended to be followed by longer durations when the same perceptual state subsequently returned.

Because short-range positive dependencies were present, we next estimated their temporal span using a parametric history kernel fitted to the empirical lag profile. We fit two standard forms—a short-memory exponential decay kernel and a power-law kernel that allows heavier tails. Within the informative window (lags 1–3), both kernels were similarly supported by the data (exponential vs power-law: AIC = 4,590 vs 4,592). For clarity, we therefore reported the exponential kernel in **Supplementary Figure 2B**. The estimated half-life of the history effect was 1.46 recurrences in lag units (approximately three switches), corresponding to 8.85 s on the time axis (**Supplementary Figure 2C**). Thus, residual dependence decayed within a few returns of the same perceptual state.

Finally, we tested whether sensory processing sensitivity moderated these history effects. Adding sensory processing sensitivity as a moderator to either the free-lag model or the kernel-based history regressor did not yield a reliable improvement in model fit. In the exponential-kernel model, the interaction between the history regressor and sensory processing sensitivity was close to zero ($\beta$ = −0.009, SE = 0.011, 95% Wald CI [−0.03, 0.01]). Taken together, switching deviated modestly from a strictly memoryless renewal process, but the dependence was short-lived and not detectably modulated by sensory processing sensitivity. These findings support the interpretation that effects of sensory processing sensitivity primarily reflect differences in state maintenance dynamics and duration distributions rather than differences in short-range sequential dependencies.

## Supplementary References


1 Murata, T., Matsui, N., Miyauchi, S., Kakita, Y. & Yanagida, T. Discrete stochastic process underlying perceptual rivalry. *Neuroreport* **14**, 1347-1352, doi:10.1097/01.wnr.0000077553.91466.41 (2003).

2 Cao, R., Pastukhov, A., Mattia, M. & Braun, J. Collective activity of many bistable assemblies reproduces characteristic dynamics of multistable perception. *J. Neurosci.* **36**, 6957-6972, doi:10.1523/jneurosci.4626-15.2016 (2016).

3 Pastukhov, A. & Braun, J. Cumulative history quantifies the role of neural adaptation in multistable perception. *J. Vis.* **11**, 1-10, doi:10.1167/11.10.12 (2011).

4 Fründ, I., Wichmann, F. A. & Macke, J. H. Quantifying the effect of intertrial dependence on perceptual decisions. *J. Vis.* **14**, 1-16, doi:10.1167/14.7.9 (2014).

**Supplementary Table 1 | Posterior summaries of time-resolved state contrast in termination probability**

| Midpoint time (s) | Mean (Δhaz) | Lower 95% CrI | Upper 95% CrI | *P*(Δhaz < 0) |
|---|---|---|---|---|
| HSP (z) = −1 SD | | | | |
| 0.5 | −0.000855 | −0.001714 | −0.000058 | 0.981 |
| 2 | 0.000690 | −0.000961 | 0.002355 | 0.200 |
| 4 | 0.001587 | −0.000441 | 0.003701 | 0.062 |
| 8 | −0.000878 | −0.003274 | 0.001452 | 0.766 |
| 12 | −0.001729 | −0.004575 | 0.000941 | 0.895 |
| 16 | −0.001363 | −0.004796 | 0.001955 | 0.786 |
| 20 | 0.000402 | −0.004019 | 0.004974 | 0.437 |
| HSP (z) = 0 (sample mean) | | | | |
| 0.5 | −0.001078 | −0.001823 | −0.000371 | 0.998 |
| 2 | −0.000111 | −0.001524 | 0.001279 | 0.561 |
| 4 | 0.000205 | −0.001427 | 0.001834 | 0.402 |
| 8 | −0.002171 | −0.004096 | −0.000312 | 0.989 |
| 12 | −0.002922 | −0.005336 | −0.000641 | 0.994 |
| 16 | −0.002712 | −0.005633 | 0.000124 | 0.969 |
| 20 | −0.001378 | −0.005290 | 0.002551 | 0.754 |
| HSP (z) = +1 SD | | | | |
| 0.5 | −0.001270 | −0.002094 | −0.000562 | 0.999 |
| 2 | −0.000797 | −0.002370 | 0.000684 | 0.850 |
| 4 | −0.000976 | −0.002974 | 0.001072 | 0.832 |
| 8 | −0.003280 | −0.005718 | −0.000967 | 0.997 |
| 12 | −0.003946 | −0.006671 | −0.001377 | 0.999 |
| 16 | −0.003869 | −0.007109 | −0.000794 | 0.994 |
| 20 | −0.002903 | −0.007102 | 0.001143 | 0.918 |

Per-bin termination probability (100-ms bins) between two-stream and one-stream percepts is defined as $\Delta\text{haz}(t) = \text{haz}_{\text{two}}(t) - \text{haz}_{\text{one}}(t)$.

Estimates are shown at prespecified time points (in seconds since phase onset).

Negative values indicate a lower termination probability (greater stabilization) for the two-stream percept relative to the one-stream percept.

HSP, Highly Sensitive Person Scale score; CrI, credible interval.

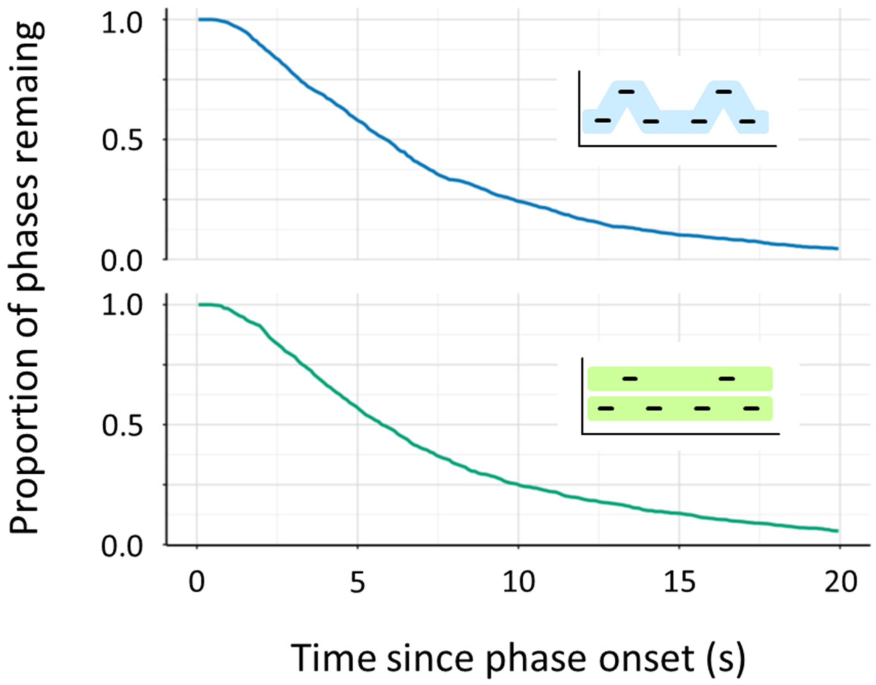


**Supplementary Figure 1 | Survival curves as a function of elapsed time since phase onset.** The curves are shown separately for two perceptual states ($N$ = 48). The top panel (blue) corresponds to one-stream phases, and the bottom panel (green) corresponds to two-stream phases. This visualization makes the size of the "at-risk" set transparent for the hazard analysis: later time points are supported by progressively fewer long phases, which motivates cautious interpretation of late-bin divergences in termination probability.

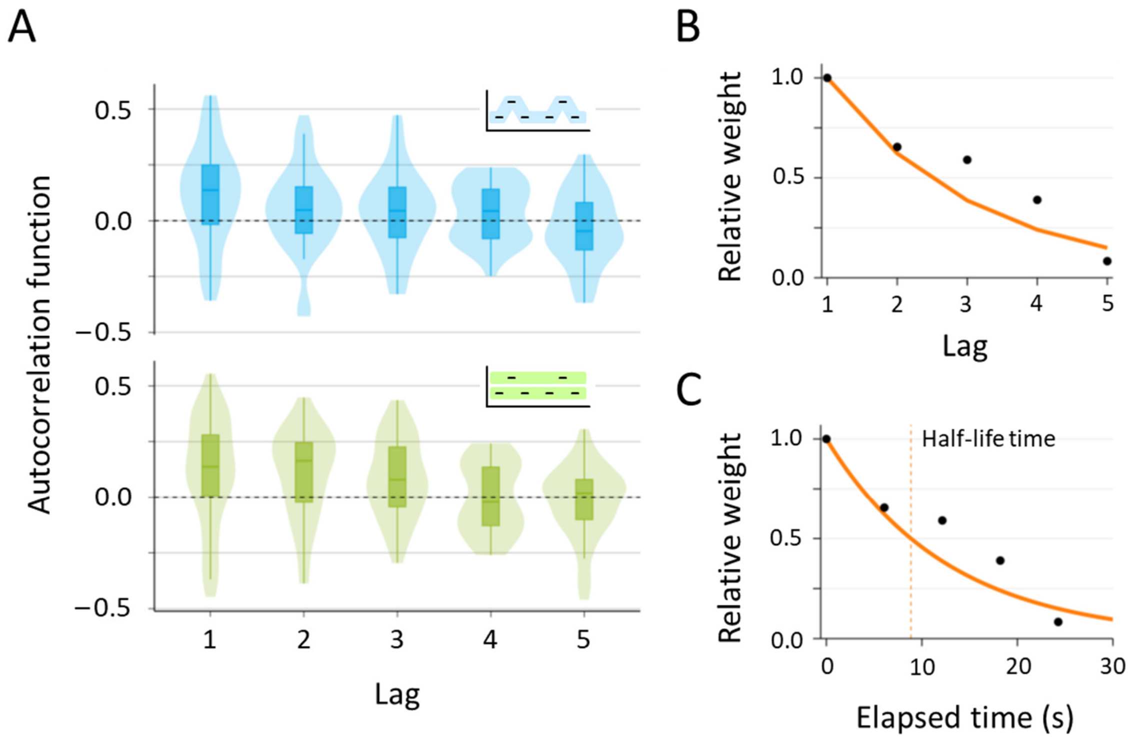


**Supplementary Figure 2 | History effects in percept durations: autocorrelation, kernel fits, and half-life.** (A) Within-state autocorrelation of log-transformed percept durations across lags 1–5. Violin plots show participant distributions (dashed line = 0). Top, one-stream (blue); bottom, two-stream (green). Positive $\rho_1$ in both states indicates short-range carry-over, which weakens with lag. (B) Free-lag regression relative weights (black dots) with exponential kernel fit to empirical lag profile. Relative weight denotes influence of a prior phase at lag *g*, normalized to lag-1 influence. (C) The same kernel expressed as a function of elapsed time (lags converted using the median percept duration), with weights normalized such that $w(1) = 1$. The vertical dashed line marks the half-life (8.85 s).
