# Supplementary material for: Sensory processing sensitivity is associated with state-dependent stabilization of perceptual organization in auditory streaming
Source: Commun Psychol. 2026 Jun 9;4:99. doi: 10.1038/s44271-026-00482-z (PMC13319501; doi:10.1038/s44271-026-00482-z)
Supplement: Supplementary file 1 — Transparent Peer Review file [file 44271_2026_482_MOESM1_ESM.pdf]

nature portfolio

Peer Review File

Sensory processing sensitivity is associated with state-dependent stabilization of perceptual organization in auditory streaming

Corresponding Author: Professor Hirohito Kondo

This file contains all editorial decision letters in order by version, followed by all author rebuttals in order by version.

Version 0:

Decision Letter:

** Please ensure you delete the link to your author homepage in this e-mail if you wish to forward it to your coauthors **

Dear Professor Kondo,

Thank you for your patience during the peer-review process. Your manuscript titled "Sensory processing sensitivity shapes the temporal dynamics of auditory bistability" has now been seen by 3 reviewers, and I include their comments at the end of this message. They find your work of interest but raised some important points. We are interested in the possibility of publishing your study in Communications Psychology, but would like to consider your responses to these concerns and assess a revised manuscript before we make a final decision on publication.

We therefore invite you to revise and resubmit your manuscript, along with a point-by-point response to the reviewers. Please highlight all changes in the manuscript text file.

Editorially, we consider it important that you address all reviewer concerns, especially the shared concerns regarding unresolved alternative interpretations of the results, the high technical complexity of the report, and the lack of clarity and sufficient detail in the justification of the modeling assumptions.

As you revise the manuscript in response to these issues, please also implement all requests in the attached Mandatory Revision Requests document. All requirements listed in this document need to be fully met, or the work will be returned to you for further revisions without peer review. This workflow is in place to increase the likelihood that the paper will be accepted for publication. It reduces the number of rounds of revision (and review) and ensures that the reviewers vet a version of the article that is compliant with journal policies. If you have any questions regarding the required revisions, please contact the journal prior to resubmission to avoid a negative outcome.

We are committed to providing a fair and constructive peer-review process. Please don't hesitate to contact us if you wish to discuss the revision in more detail.

Please submit the following items:

- Revised manuscript
- Point-by-point response to the referees’ comments
- Mandatory Revision Requests Table (attached).
- Cover letter (as a separate document)

via this link: Link Redacted .

** This url links to your confidential home page and associated information about manuscripts you may have submitted or are reviewing for us. If you wish to forward this email to co-authors, please delete the link to your homepage first **

We hope to receive your revised paper within 8 weeks; please let us know if you aren't able to submit it within this time so that we can discuss how best to proceed. If we don't hear from you, and the revision process takes significantly longer, we may close your file. In this event, we will still be happy to reconsider your paper at a later date, provided it still presents a significant contribution to the literature at that stage.

We look forward to seeing the revised manuscript and thank you for the opportunity to review your work.

Best regards,

David Pascucci

David Pascucci
Guest Editor
Communications Psychology

REVIEWER REPORTS:

Reviewer #1 (Remarks to the Author):

This is a very interesting and well-executed individual differences study linking sensory processing sensitivity (SPS; HSP scale) to auditory bistability. The main empirical result (higher SPS associated with longer dominance durations end fewer switches) seems robust at the descriptive level. The attempt to localize the effect using multiple, interpretable modeling approaches is a strength. Overall, I feel that the manuscript if a great candidate for publication in Communications Psychology.

My main concern is that several aspects currently leave open alternative interpretations that are not fully addressed in the current report.

Best wishes,
Veith Weilnhammer

Major

1) The authors interpret higher SPS as "perceptual stability". Yet SPS could maybe also correlate with non-perceptual cognitive functions, such as reporting behavior or attention (e.g., response caution, ability to focus on the experiment, compliance with continuous reporting). It is at least a possibility that inter-individual differences among those functions could also lengthen reported dominance durations and reduce switch rate.
I would suggest that the authors report and discuss explicitly whether SPS correlates with measures that index reporting/executive behavior (e.g., frequency of "mixed/uncertain" reports if available, response times, etc.). If such measures are unavailable, I feel that the authors should acknowledge the limitation more directly and consider adding addition control analyses (e.g., how robust are the results to excluding participants above the 95% percentile in dominance duration, or below the 5% percentile in dominance duration?).

2) The recurring asymmetries between one-stream and two-stream states suggests that a bias toward the two-stream state may contribute to the findings. The manuscript reports mainly state-specific effects. Critically, the association of SPS differs between the two states. At present, one can reasonably ask whether SPS primarily increases time in (or bias toward) two-stream, with the reduced switching rate following secondarily.
I would therefore suggest conducting all analyses that are, at present, done in isolation for each state, across both states. If there is a clear reason for not doing this, which I may have missed, then this should be very clearly reported in the manuscript. I would also suggest that the authors test more directly whether SPS predicts occupancy/bias (proportion time in each state) independently of effects on switching dynamics.

3) The DDM analysis focuses on drift, boundary, and non-decision time. Yet in standard DDM applications, starting point (z) is a key parameter capturing pre-decisional bias toward a choice. Given the evident direction/state asymmetry, omitting z leaves a major explanatory degree of freedom untested. I would therefore suggest fitting the starting point as well and test whether SPS relates to z (and whether including z changes the apparent v/t0 effects). If there is a reason for not doing that, the authors should make this case very clear.

4) The hazard result is described as an SPS effect "right after a percept became dominant," yet the plotted differences appear emphasized later (that is, significance around 8–20 s). Given that median dominance durations are around 6 s, time points beyond around 12 s likely reflect a shrinking subset of long phases. Without showing how much data contribute to each timepoint in the analysis, the interpretation is hard to evaluate. I would suggest plotting the fraction of phases still

ongoing curve beneath the hazard plot, to clarify how much information supports differences later in time. I am also confused by the "right after a percept became dominant" wording, since the curves overlap initially.

5) Given the strong mechanistic interpretation placed on parameters (drift, boundary, non-decision time), the manuscript would benefit from a parameter recovery analysis. One could define a grid of plausible parameter sets (including starting point if added), simulate datasets matching to the empirical amount of data, refit the model, and report recovery (correlation/bias) for each parameter.

Minor:

6) The "gamma first-passage" section is hard to follow for non-specialist readers. Maybe it would be helpful to explain what we learn from it in simpler terms for the uninitiated reader.

7) Figure 1B: The text states that after a brief transient, probability fluctuates around 0.5 with derivative near zero, implying stable bistability. Visually, it is not obvious that there is no slow drift (and the value at time 0 is unclear, with upper plot near 0 while lower starts near 0.5). This may simply reflect smoothing, initialization, or how the first sample is handled? Can the authors comment on and test slow drift?

8) The manuscript could benefit from relating this work more explicitly to predictive-processing literature on bistable perception, given its previous applications to switching dynamics, inter-individual differences in psychological function, serial dependence, and drift diffusion.

Reviewer #2 (Remarks to the Author):

The present manuscript by Kondo and Pressnitzer investigates whether sensory processing sensitivity (SPS; measured by the Highly Sensitive Person scale) is associated with perceptual switching and dominance dynamics in an auditory streaming bistability paradigm, using continuous reports of one-stream versus two-stream percepts. The manuscript reports that higher SPS is associated with fewer switches and longer dominance durations, and it attempts to localize candidate mechanisms via DDM-based switching analyses, discrete-time hazard modeling, and distributional decompositions of dominance times, with additional analyses of short-range history dependence. The study is interesting in its concept, but several core issues must be resolved before the conclusions can be evaluated.

Major:

1. The manuscript applies the DDM by treating each switch as an independent perceptual decision and introduces a standard two-choice DDM with absorbing boundaries (±a) reached by the decision variable x(t). However, in a continuous bistable stream there is no discrete trial onset, and after a switch the system is (by definition) in a stable perceptual state. Under a literal mapping where the boundaries correspond to the two percepts, the post-switch state would place x(t) at/near a boundary, which would imply implausibly short subsequent decision times in a standard absorbing-boundary DDM unless an additional reset/re-initialization rule is explicitly assumed.

At present, the manuscript does not clearly specify 1) what latent quantity x(t) represents in this bistable context, 2) what the boundaries correspond to, and 3) what the initial condition is at the onset of each dominance phase (and how it is set after a switch). Relatedly, because the analyses treat one→two and two→one durations separately, the implementation reads more like fitting two state-dependent one-boundary first-passage/hazard processes (time-to-leave each perceptual state) rather than a single two-choice DDM in which "choice" is determined by which boundary is hit.

This issue is particularly important given that the manuscript cites prior computational accounts of bistable auditory streaming that include both competition–adaptation dynamics and evidence-accumulation formulations (e.g., Rankin et al., 2015; Nguyen et al., 2020), where generative assumptions are typically made explicit (what is accumulated, whether accumulation saturates, and how the state/accumulator is re-initialized after a switch). Please clarify the intended generative mapping and, if appropriate, reframe the model explicitly as a one-boundary diffusion/hazard (state-leaving) model, or otherwise provide a principled justification and explicit assumptions (starting point/reset rule) that make a two-boundary DDM coherent for continuous bistable switching.

2. The central interpretation ("higher SPS stabilizes perception") is not yet coherently integrated with the direction-specific modeling results. As written, the evidence for fewer switches and longer dominance periods overall, together with the hazard-based claim of a reduced termination risk, is not clearly reconciled with the DDM results that emphasize direction-specific SPS associations in the fitted parameters. The manuscript would benefit from a clearer conceptual decomposition of what "stability" means here (e.g., tendency to enter a perceptual state vs. tendency to maintain it once established), and from making explicit how the DDM-based account maps onto these components.

Please provide model checks demonstrating that the fitted switching model (with SPS-dependent parameters) reproduces the key summaries used in the manuscript (switch counts, state occupancy/time spent in each percept, and state-specific duration distributions), so that the DDM serves as an explanatory link rather than an additional layer of post hoc interpretation.

Relatedly, the manuscript reports many outcomes and parameter-level associations across multiple analytic frameworks, which increases the risk of selective emphasis and inflated false positives even when some corrections are applied locally. It would help to clarify which outcome(s) are central to the main claim, and how the remaining analyses could be interpreted (supporting/exploratory). Please also state clearly how multiple testing was handled across these result families.

3. The Introduction places SPS/HSP within a broad "environmental sensitivity" framework and related literature, but the empirical contribution is narrowly centered on bistable auditory streaming dynamics and parameter interpretations within that paradigm. As currently written, the framing can read as "many traits covary with bistability, so we also tested HSP," rather than a hypothesis-driven link. Please tighten the narrative by 1) adding a more specific mechanistic prediction connecting SPS to auditory scene analysis/stream segregation, and 2) keeping broader claims about environmental sensitivity proportional to what can be supported by a single bistable streaming task.

4. The manuscript introduces "serial dependence" rather suddenly, in a way that can feel disconnected from the main modeling narrative and may be confusing given how strongly the term is associated with perceptual/decisional bias phenomena in the recent literature. In this paper, the relevant issue appears to be a methodological assumption: the models treat switches as independent events in a renewal process and the authors then test for sequential/history dependence in dominance durations (autocorrelations; parametric history kernels) and whether SPS moderates it. I recommend reframing the serial dependence section explicitly as a robustness check of the renewal/independence assumption (e.g., history dependence / sequential dependence of dominance durations, etc.), rather than presenting it under the banner of serial dependence, to reduce the risk of readers interpreting the section as addressing the broader serial-dependence literature.

5. Alternative explanations linked to attention and response strategy need to be addressed more directly. With long continuous reporting, lower switch rates can reflect conservative criteria, delayed reporting, fatigue, or compliance differences rather than perceptual dynamics per se, and SPS may correlate with such factors as well as with anxiety traits, musical experience, or auditory acuity. Please expand the limitations accordingly and, if any relevant measures are available, report them; if not, consider simple robustness checks to rule out the most obvious reporting-strategy artifacts (e.g., participants with extremely low switch counts or evidence of prolonged unresponsive periods).

Minor:

1. The hazard-model description is inconsistent with respect to temporal binning and the reported quantity. In different parts of the manuscript the hazard is described in incompatible units (e.g., 100-ms bins vs 1-s intervals), which makes it difficult to reproduce the analysis as written. Please state the bin width consistently, define whether the dependent variable is a per-bin event probability or a rate, and describe the time basis functions/smoothing and covariate coding in a way that matches what is plotted and reported.

Relatedly, in Fig. 4, the shaded regions are not clearly defined in the legend (e.g., whether they represent bin widths, uncertainty bands, or highlighted evaluation windows). As currently presented, the shading can be misread as indicating a continuous time interval over which the hazard difference is significant, even with the additional "ns" labels. Please explicitly describe what the shaded regions represent in the legend, and align the visualization with the discrete-time/binning and selected evaluation-time testing procedure (e.g., clarify that significance is assessed only at the pre-specified time points with Holm adjustment).

2. Please add any missing methodological details needed for replication, including audio presentation details (calibration/level, device/headphones, sampling rate, etc.), and clarify instructions about how participants should report during ambiguous or transitional percepts.

3. The statement “In an FPT framework, by contrast, a switch is described as the completion of a small number of discrete sub-events” (ll. 255–258) is misleading. A standard DDM is itself an FPT model (diffusion-to-bound), and the manuscript elsewhere contrasts diffusion-to-bound (inverse-Gaussian waiting times) with discrete sub-event accumulation (Gamma/Erlang). Please revise the phrasing to reflect that the FPT perspective is a broader umbrella (including diffusion-to-bound/DDM) and that the discrete sub-event account is one candidate mechanism within that perspective, rather than its definition.

Also, the phrasing “each switch is treated as continuous accumulation” is imprecise. In a DDM, the switch is a boundary-crossing (first-passage) event, whereas the continuous accumulation describes the latent process during the interval between switches.

Reviewer #3 (Remarks to the Author):

Summary

The authors used a number of modeling approaches to estimate the impact of Sensory Processing Sensitivity (SPS), per the Highly Sensitive Person (HSP) scale, on auditory stream processing. Their paradigm showed a balanced bistable effect, whereby participants reported hearing one and two streams with relatively equal probability. Individuals with higher SPS perceived auditory streams for longer durations, with fewer switches between streams and greater stability (in the 8-20s window after percept onset), than lower SPS individuals. Additionally, HSP scores were associated with a tendency towards

perceiving segregated streams, and that switching from segregated to integrated streams involved fewer, noisier decision steps.

Major Feedback

Overall, the paper tests an interesting question about the interaction of external stimuli and individual traits that influence endogenous processing, and discusses the use of models in future research, thus adding to the field. However, the paper is highly technical, with several core concepts not fully defined, limiting the paper's accessibility. Consider explaining concepts such as exploration (in the context of ego-resiliency), sub-events, direction-specific entry, for readers not in your immediate field and use more plain language in your initial summary of the results in the Discussion.

The authors mention the models are meant to be complementary, but there seem to be a number of measures capturing aspects of switch propensity that are likely related and sharing variance. The paper would benefit from additional discussion of what potential mechanisms might explain the HSP effects on drift and hazard but not k.

I appreciate the discussion around serial dependencies and independence assumptions in bistable perception models, yet I wonder about the DDM implementation. While HSP doesn't modulate autocorrelation, autocorrelation still exists. Why not use an autoregressive (AR1) DDM structure? Additionally, was the interaction between switch direction and autocorrelation tested?

Minor Feedback

- Consider more clearly introducing ABA-tone sequence, for those unfamiliar (e.g. "the classic sequence of triple tones, ABA, listeners …")
- Line 68-9, do authors mean to write "to treat switches AS perceptual decisions"?
- Fig 3 caption, Line 192, very minor but it would read more smoothly to give a little more context in the caption where it reads (B) As (A) (e.g. (B) As above, or Similar to panel (A))
- Fig. 4 should have a legend for the different lines, not just written in the caption.

* TRANSPARENT PEER REVIEW: Communications Psychology uses a transparent peer review system. This means that we publish the editorial decision letters including Reviewers' comments to the authors and the author rebuttal letters online as a supplementary peer review file. However, on author request, confidential information and data can be removed from the published reviewer reports and rebuttal letters prior to publication. If your manuscript has been previously reviewed at another journal, those Reviewers' comments would not form part of the published peer review file.

** Visit Nature Research's author and referees' website at www.nature.com/authors for information about policies, services and author benefits**

Communications Psychology is committed to improving transparency in authorship. As part of our efforts in this direction, we are now requesting that all authors identified as 'corresponding author' create and link their Open Researcher and Contributor Identifier (ORCID) with their account on the Manuscript Tracking System prior to acceptance. ORCID helps the scientific community achieve unambiguous attribution of all scholarly contributions. You can create and link your ORCID from the home page of the Manuscript Tracking System by clicking on 'Modify my Springer Nature account' and following the instructions in the link below. Please also inform all co-authors that they can add their ORCIDs to their accounts and that they must do so prior to acceptance.
https://www.springernature.com/gp/researchers/orcid/orcid-for-nature-research

For more information please visit http://www.springernature.com/orcid

If you experience problems in linking your ORCID, please contact the Platform Support Helpdesk.

Version 1:

Decision Letter:

** Please ensure you delete the link to your author homepage in this e-mail if you wish to forward it to your coauthors **

Dear Professor Kondo,

Your manuscript titled "Sensory processing sensitivity is associated with state-dependent stabilization in auditory streaming" has now been seen by our reviewers, whose comments appear below. In light of their advice I am delighted to say that we are happy, in principle, to publish a suitably revised version in Communications Psychology.

We therefore invite you to revise your paper one last time to address the remaining concerns of our reviewers and a list of editorial requests. At the same time we ask that you edit your manuscript to comply with our format requirements and to maximise the accessibility and therefore the impact of your work.

EDITORIAL REQUESTS:

Please review our specific editorial comments and requests regarding your manuscript in the attached "Editorial Requests Table". Please outline your response to each request in the right hand column. Please upload the completed table with your manuscript files as a Related Manuscript file.

If you have any questions or concerns about any of our requests, please do not hesitate to contact me.

SUBMISSION INFORMATION:

In order to accept your paper, we require the files listed here https://www.nature.com/documents/commsj-file-checklist.pdf .

OPEN ACCESS:

Communications Psychology is a fully open access journal. Articles are made freely accessible on publication. For further information about article processing charges, open access funding, and advice and support from Nature Research, please visit https://www.nature.com/commspsychol/open-access

At acceptance, you will be provided with instructions for completing the open access licence agreement on behalf of all authors. This grants us the necessary permissions to publish your paper. Additionally, you will be asked to declare that all required third party permissions have been obtained, and to provide billing information in order to pay the article-processing charge (APC).

* TRANSPARENT PEER REVIEW: Communications Psychology uses a transparent peer review system. On author request, confidential information and data can be removed from the published reviewer reports and rebuttal letters prior to publication. If you are concerned about the release of confidential data, please let us know specifically what information you would like to have removed. Please note that we cannot incorporate redactions for any other reasons.

* CODE AVAILABILITY: All Communications Psychology manuscripts must include a section titled "Code Availability" at the end of the methods section. We require that the custom analysis code supporting your conclusions is made available in a publicly accessible repository at this stage; please choose a repository that generates a digital object identifier (DOI) for the code; the link to the repository and the DOI must be included in the Code Availability statement. Publication as Supplementary Information will not suffice.

* DATA AVAILABILITY:
All Communications Psychology manuscripts must include a section titled "Data Availability" at the end of the Methods section. More information on this policy, is available in the Editorial Requests Table and at http://www.nature.com/authors/policies/data/data-availability-statements-data-citations.pdf.

Please use the following link to submit the above items:
Link Redacted
** This url links to your confidential home page and associated information about manuscripts you may have submitted or be reviewing for us. If you wish to forward this email to co-authors, please delete the link to your homepage first **

We hope to hear from you within two weeks; please let us know if you need more time.

Best regards,

Marike, on behalf of David Pascucci

Marike Schiffer, PhD
Chief Editor
Communications Psychology

REVIEWERS' COMMENTS:

Reviewer #1 (Remarks to the Author):

The authors have been very responsive to my comments.

Reviewer #2 (Remarks to the Author):

The authors have addressed all of my previous comments adequately.

** Visit Nature Research's author and referees' website at www.nature.com/authors for information about policies, services and author benefits**

Open Access This Peer Review File is licensed under a Creative Commons Attribution 4.0 International License, which permits use, sharing, adaptation, distribution and reproduction in any medium or format, as long as you give appropriate credit to the original author(s) and the source, provide a link to the Creative Commons license, and indicate if changes were made.
In cases where reviewers are anonymous, credit should be given to 'Anonymous Referee' and the source.
The images or other third party material in this Peer Review File are included in the article’s Creative Commons license, unless indicated otherwise in a credit line to the material. If material is not included in the article’s Creative Commons license and your intended use is not permitted by statutory regulation or exceeds the permitted use, you will need to obtain permission directly from the copyright holder.
To view a copy of this license, visit https://creativecommons.org/licenses/by/4.0/

Dear Editor,

We would like to sincerely thank you and the three reviewers for their detailed, thoughtful, and constructive feedback on the first version of the manuscript. We believe that the present revision addresses all comments, as detailed in the rebuttal below. For ease of review, changes in the revised manuscript are indicated in brick red.

The most substantial revision concerns our previous use of a drift-diffusion model (DDM) to account for switching dynamics. All three reviewers identified several issues with this idea: the underlying decision variable was not clearly specified, so there was an ambiguity in the meaning of the boundaries. Moreover, additional assumptions about starting point, reset, and bias would be needed to explicitly map the model to putative underlying mechanisms. We agree that these concerns were well founded and sufficiently serious that we removed the DDM analysis entirely. Instead, we focused the behavioral data analysis on three interpretable approaches, addressing distinct facets of the generic idea of "perceptual stability": (i) overall state occupancy; (ii) time-resolved state maintenance; and (iii) distributional properties of dominance durations. Concretely, we used a Bayesian beta-regression occupancy analysis, which allowed us to introduce further robustness checks; a discrete-time hazard model of phase termination; and a Gamma distributional regression that decomposes dominance durations into timescale and regularity/shape components. The resulting associations with SPS are broadly consistent with our original interpretation, but they are now more clearly motivated, more systematically documented, and more robustly supported.

Second, prompted in part by comments of Reviewer #1, we considered in depth the framing of our findings in terms of predictive theories of perceptual organization. This turned out to be a fruitful idea: a recent review of sensory processing sensitivity (Greven et al., 2025) proposes that the SPS trait may be understood in terms of atypically high precision assigned to sensory evidence. Because predictive theories have also proposed and tested for auditory streaming (e.g., Bendixen et al., 2010), we can now be much more specific in how SPS may impact auditory scene analysis. The Discussion has been extensively reorganized to reflect this perspective, while keeping the interpretation appropriately cautious.

Numerous other changes were made in order to try and improve clarity, reproducibility, and the strength of the inferences. First, we updated the title to more accurately reflect the main conclusion. Second, to enhance transparency and facilitate replication, we deposited the full dataset and analysis scripts in an Open Science Framework (OSF). Finally, we revised the manuscript to comply with the journal's Mandatory Revision Requests, including reorganizing and expanding methodological reporting where needed (e.g., Bayesian-analysis reporting details and consistent hazard-model binning and definitions).

Overall, these revisions were intended to address alternative explanations more directly, reduce unnecessary technical burden where possible, and provide a clearer and more reproducible account of how SPS relates to the microdynamics of auditory bistability. We believe that they have substantially strengthened the manuscript, and we would like to thank the reviewers again for their many valuable insights.

Reviewer #1

Comment: *This is a very interesting and well-executed individual differences study linking sensory processing sensitivity (SPS; HSP scale) to auditory bistability. The main empirical result (higher SPS associated with longer dominance durations end fewer switches) seems robust at the descriptive level. The attempt to localize the effect using multiple, interpretable modeling approaches is a strength. Overall, I feel that the manuscript if a great candidate for publication in Communications Psychology.*

My main concern is that several aspects currently leave open alternative interpretations that are not fully addressed in the current report.

Best wishes,

Veith Weilnhammer

Response: Thank you very much for your overall positive evaluation. We are glad that the main individual-differences finding was seen as robust and that the multi-model approach was considered a strength. Below we address each of your helpful insights and indicate changes in the revised manuscript.

Major

1) The authors interpret higher SPS as "perceptual stability". Yet SPS could maybe also correlate with non-perceptual cognitive functions, such as reporting behavior or attention (e.g., response caution, ability to focus on the experiment, compliance with continuous reporting). It is at least a possibility that inter-individual differences among those functions could also lengthen reported dominance durations and reduce switch rate.

I would suggest that the authors report and discuss explicitly whether SPS correlates with measures that index reporting/executive behavior (e.g., frequency of "mixed/uncertain" reports if available, response times, etc.). If such measures are unavailable, I feel that the authors should acknowledge the limitation more directly and consider adding addition control analyses (e.g., how robust are the results to excluding participants above the 95% percentile in dominance duration, or below the 5% percentile in dominance duration?).

We fully agree that longer reported durations and the accompanying fewer switches could, in principle, reflect non-perceptual factors such as the ones you mention. Our task did not include an option for participants to report "mixed/uncertain" epochs, as instead, we used the common two-alternative continuous reporting paradigm (one-stream vs two-stream). Thus, we cannot directly test SPS associations with the frequency of mixed reports. However, we now explicitly acknowledge this limitation.

We further appreciate your suggestion to assess robustness via excluding extreme participants (e.g., above the 95th or below the 5th percentile). We have not implemented it for now, though, as it could

be argued that defining a somewhat arbitrary threshold to remove participants may inadvertently discard meaningful individual-difference variance. Instead, but to address the same valid concern, we have shifted the revised analyses toward a Bayesian, model-based framework. This framework can quantify uncertainty directly and evaluate the stability of SPS effects across multiple complementary models. In particular, we used Bayesian beta regression for state occupancy, so that the central conclusions are supported by converging evidence that does not hinge on any exclusion rule. We hope you will find this new robustness check convincing. Otherwise, we could of course implement the exclusion method in a further revision, if necessary.

2) The recurring asymmetries between one-stream and two-stream states suggests that a bias toward the two-stream state may contribute to the findings. The manuscript reports mainly state-specific effects. Critically, the association of SPS differs between the two states. At present, one can reasonably ask whether SPS primarily increases time in (or bias toward) two-stream, with the reduced switching rate following secondarily.

You are correct, the alternative interpretation that you point out, in which SPS affects state occupancy, could not be ruled out by the analyses reported in the previous version of the manuscript. In the revision, we therefore tested this possibility directly, by modeling two-stream occupancy (the proportion of total reporting time spent in the two-stream state) using Bayesian beta regression. The posterior provided little evidence that higher SPS shifted the overall balance toward segregation. This crucial but perhaps counter-intuitive finding is further addressed in the “Limitations” subsection of the Discussion.

I would therefore suggest conducting all analyses that are, at present, done in isolation for each state, across both states. If there is a clear reason for not doing this, which I may have missed, then this should be very clearly reported in the manuscript. I would also suggest that the authors test more directly whether SPS predicts occupancy/bias (proportion time in each state) independently of effects on switching dynamics.

Thank you for the helpful suggestion, which we implemented. The revised inferential analyses now include both states at once. In the discrete-time hazard model, perceptual state and its interaction with SPS are included within a single model, allowing us to estimate state-dependent effects on termination dynamics directly, which is much more elegant and convincing. This model, in line with the previous analyses, indicates that SPS was more negatively associated with termination probability in the two-stream state than in the one-stream state. This is crucial to avoid resting our claim of state-dependence on null results.

Lines 329–334:

> The main effect of sensory processing sensitivity on termination within the one-stream state

was small and uncertain (β_{hsp} = −0.03, 95% CrI [−0.19, 0.11]). However, sensory processing sensitivity showed a negative association with termination in the two-stream state, as reflected in the interaction term (β_{int} = −0.09, 95% CrI [−0.18, −0.00]). This pattern is consistent with the interpretation that greater sensory processing sensitivity was associated with enhanced maintenance of the two-stream state once established.

Finally, our Gamma distributional regression likewise evaluates state dependence within a unified framework and indicates that the SPS association is most clearly expressed in the dominance durations for two-stream phases. Taken together, the analyses clarify the general concept of "perceptual stability" into (i) overall occupancy (bias/entry) versus (ii) state maintenance. Results show that the reduced switching observed at higher SPS is unlikely to be explained simply by overall occupancy, but instead derives from state maintenance processes.

3) The DDM analysis focuses on drift, boundary, and non-decision time. Yet in standard DDM applications, starting point (z) is a key parameter capturing pre-decisional bias toward a choice. Given the evident direction/state asymmetry, omitting z leaves a major explanatory degree of freedom untested. I would therefore suggest fitting the starting point as well and test whether SPS relates to z (and whether including z changes the apparent v/t0 effects). If there is a reason for not doing that, the authors should make this case very clear.

We thank you for raising this important point, which relates to remarks from the other reviewers too. With hindsight, we agree the DDM framework does not map as easily as we implied to the perceptual bistability task. Indeed, we would need to make additional assumptions on starting-point bias, reset mechanisms, and the mapping between decision bounds and perceptual transitions. A possible solution, inspired by your work, would be to consider that the accumulated variable is the prediction error associated to the current perceptual interpretation. This assumption and accompanying parameters would need to be made up from scratch, though, providing us with a lot of degrees of freedom.

Given that all the reviewers objected to some extent to the DDM analysis, and given that we can support the same main conclusions with the other analyses, we therefore removed the DDM entirely from the manuscript. Hopefully we will be able to evaluate the applicability of such a framework to auditory streaming in a future, more focused contribution, but for now we take the point that our treatment was unsatisfactory.

4) The hazard result is described as an SPS effect "right after a percept became dominant," yet the plotted differences appear emphasized later (that is, significance around 8–20 s). Given that median dominance durations are around 6 s, time points beyond around 12 s likely reflect a shrinking subset

of long phases. Without showing how much data contribute to each timepoint in the analysis, the interpretation is hard to evaluate. I would suggest plotting the fraction of phases still ongoing curve beneath the hazard plot, to clarify how much information supports differences later in time. I am also confused by the "right after a percept became dominant" wording, since the curves overlap initially.

We agree that our wording was misleading. In the revised manuscript, we now describe the result quantitively as a time-resolved, state-dependent modulation of phase-termination probability, with differences emerging primarily later approximately 8–20 s after phase onset.

We also agree with the concern that estimates at longer elapsed times are necessarily based on a progressively smaller subset of longer phases, leading to less supporting evidence. To avoid over-interpreting this tail region, we now (i) explicitly acknowledge in the text that the later time bins reflect fewer remaining phases, and (ii) emphasize that our inference is anchored in the model-based estimate of the hazard function rather than in any single late time point. In response to the reviewer's suggestion, we have clarified in the figure caption/text that the hazard curves overlap early in the phase and that the divergence is not an "immediate post-onset" effect.

Lines 348–351:

> For average and greater sensory processing sensitivity, Δhaz(t) became credibly negative in the later portion of the dominance phase, indicating lower termination probability, i.e., greater stabilization, for the two-stream percept relative to the one-stream percept (**Supplementary Table 1**).

Finally, we appreciate the suggestion to plot the fraction of phases still ongoing (i.e., a survival curve) to make the amount of evidence at each time bin transparent. Following this suggestion, we now provide the corresponding survival curves in **Supplementary Fig. 1**. This clarifies that later elapsed times are supported by a progressively smaller subset of long phases. Accordingly, we describe the late-bin divergence conservatively.

5) Given the strong mechanistic interpretation placed on parameters (drift, boundary, non-decision time), the manuscript would benefit from a parameter recovery analysis. One could define a grid of plausible parameter sets (including starting point if added), simulate datasets matching to the empirical amount of data, refit the model, and report recovery (correlation/bias) for each parameter.

We agree that a parameter recovery was a very interesting suggestion in the framework of the DDM. However, as we removed the DDM analysis altogether, the DDM parameter recovery analysis is no longer applicable.

Minor

6) The "gamma first-passage" section is hard to follow for non-specialist readers. Maybe it would be helpful to explain what we learn from it in simpler terms for the uninitiated reader.

Point taken. In the revised manuscript, we added a brief explanation of what this analysis contributes—namely, that it summarizes the distribution of dominance durations in a way that separates a timescale component (how long phases tend to last) from a regularity/shape component (how variable or heavy-tailed the durations are). We also state explicitly how this complements the hazard analysis, which in contrast focuses on local termination dynamics.

7) Figure 1B: The text states that after a brief transient, probability fluctuates around 0.5 with derivative near zero, implying stable bistability. Visually, it is not obvious that there is no slow drift (and the value at time 0 is unclear, with upper plot near 0 while lower starts near 0.5). This may simply reflect smoothing, initialization, or how the first sample is handled? Can the authors comment on and test slow drift?

Well spotted. We agree that the appearance of **Figure 1B** near time 0 and the visibility of very slow trends is influenced by initialization choices and by the Gaussian smoothing used for visualization. We have therefore clarified in the text that **Figure 1B** is mostly intended as a descriptive overview of rapid stabilization and subsequent low-amplitude fluctuations, rather than a formal stationarity test.

Following your suggestion, we tested for the possibility of slow drift directly. We used unsmoothed reports aggregated into short time windows and fitted a binomial mixed-effects model with participant-specific intercepts and slopes (reported in **Supplementary Results**). This analysis indicated a small negative time trend across the trial ($\beta = -0.143$, SE = 0.048, $z = -2.97$, $p = 0.003$), consistent with the slightly reduced two-stream reporting visible in the figure. We point out, however, that the magnitude of this drift is modest and the overall probability remains near the balanced regime at the group level.

8) The manuscript could benefit from relating this work more explicitly to predictive-processing literature on bistable perception, given its previous applications to switching dynamics, inter-individual differences in psychological function, serial dependence, and drift diffusion.

We thank the reviewer for this suggestion, which proved to be extremely useful. As it turns out, predictive processing seems to be the best common ground to discuss our experimental results. For streaming, after mentioning the theoretical and modeling work of Winkler, Denham and colleagues, we focus on the paper of Bendixen et al. 2010 (which, embarrassingly, one of us actually reviewed at the time!). Their findings were intriguingly similar to ours: acoustic regularities, used a proxy to manipulate predictability, induced a stabilization of the two-stream percept only, closely paralleling our findings pertaining to the influence of SPS. In addition, a recent review of SPS (Greven et al. 2025) actually suggests that SPS is best explained within a predictive coding framework, as an increase in

the precision ascribed to incoming sensory signals.

These two strands of evidence allow us to formulate much more specific points about a tentative mechanistic account of how SPS modulated streaming in our data, in the precise way it did. The argument is now the main focus of the Discussion ("Predictive coding in auditory streaming and sensory processing sensitivity"). Thus, thanks a lot for prodding us to revisit more systematically this highly relevant literature, which allowed us to make hopefully interesting connections we had missed in the first version of the manuscript.

Reviewer #2

The present manuscript by Kondo and Pressnitzer investigates whether sensory processing sensitivity (SPS; measured by the Highly Sensitive Person scale) is associated with perceptual switching and dominance dynamics in an auditory streaming bistability paradigm, using continuous reports of one-stream versus two-stream percepts. The manuscript reports that higher SPS is associated with fewer switches and longer dominance durations, and it attempts to localize candidate mechanisms via DDM-based switching analyses, discrete-time hazard modeling, and distributional decompositions of dominance times, with additional analyses of short-range history dependence. The study is interesting in its concept, but several core issues must be resolved before the conclusions can be evaluated.

We thank the reviewer for the careful reading and for the clear summary of the study. We appreciate the reviewer's assessment that the question is of interest, and we took very seriously the core issues raised. Below we address each point and describe the corresponding changes in the revised manuscript.

Major

1. The manuscript applies the DDM by treating each switch as an independent perceptual decision and introduces a standard two-choice DDM with absorbing boundaries (±a) reached by the decision variable x(t). However, in a continuous bistable stream there is no discrete trial onset, and after a switch the system is (by definition) in a stable perceptual state. Under a literal mapping where the boundaries correspond to the two percepts, the post-switch state would place x(t) at/near a boundary, which would imply implausibly short subsequent decision times in a standard absorbing-boundary DDM unless an additional reset/re-initialization rule is explicitly assumed.

At present, the manuscript does not clearly specify 1) what latent quantity x(t) represents in this bistable context, 2) what the boundaries correspond to, and 3) what the initial condition is at the onset of each dominance phase (and how it is set after a switch). Relatedly, because the analyses treat one→two and two→one durations separately, the implementation reads more like fitting two state-dependent one-boundary first-passage/hazard processes (time-to-leave each perceptual state) rather

than a single two-choice DDM in which "choice" is determined by which boundary is hit.

This issue is particularly important given that the manuscript cites prior computational accounts of bistable auditory streaming that include both competition–adaptation dynamics and evidence-accumulation formulations (e.g., Rankin et al., 2015; Nguyen et al., 2020), where generative assumptions are typically made explicit (what is accumulated, whether accumulation saturates, and how the state/accumulator is re-initialized after a switch). Please clarify the intended generative mapping and, if appropriate, reframe the model explicitly as a one-boundary diffusion/hazard (state-leaving) model, or otherwise provide a principled justification and explicit assumptions (starting point/reset rule) that make a two-boundary DDM coherent for continuous bistable switching.

We thank the reviewer for this detailed and convincing critique. A similar point has been raised by all three reviewers.

We agree that, as originally presented, mapping a standard two-boundary two-choice DDM onto continuous bistable switching contained unresolved ambiguities—most notably (i) the interpretation of the latent decision variable and bounds in a continuous stream, (ii) the appropriate initial condition immediately after perceptual switching, and (iii) the necessity of an explicit reset/re-initialization rule to avoid implausible post-switch behavior. We also agree that, given these unresolved assumptions, the implementation can be read as closer to state-leaving (one-boundary) time-to-exit processes than to a coherent single two-choice DDM framework. This critique led us to reconsider the appropriateness of including any DDM-based switching analysis. We concluded that the necessary additions to the model would be difficult to justify satisfactorily, without giving it undue focus relative to the full story about inter-individual differences. We therefore removed the DDM analysis entirely.

Instead, we substantially restructured the Results and Discussion around a set of complementary approaches with explicit, directly interpretable mappings to the behavioral data. Specifically, we now (i) quantify overall state occupancy, (ii) model phase termination over time using a discrete-time hazard framework, and (iii) characterize dominance-duration distributions using a Gamma distributional regression. This revised framework directly addresses state asymmetries and switching dynamics without relying on DDM-specific latent decision parameters or arbitrary reset assumptions.

We are grateful to the reviewer for highlighting these issues so clearly. Removing the DDM, we feel, helped us simplify the manuscript and improve its conceptual coherence, without changing qualitatively the main claims.

2. The central interpretation ("higher SPS stabilizes perception") is not yet coherently integrated with the direction-specific modeling results. As written, the evidence for fewer switches and longer dominance periods overall, together with the hazard-based claim of a reduced termination risk, is not clearly reconciled with the DDM results that emphasize direction-specific SPS associations in the fitted parameters. The manuscript would benefit from a clearer conceptual decomposition of what "stability"

means here (e.g., tendency to enter a perceptual state vs. tendency to maintain it once established), and from making explicit how the DDM-based account maps onto these components.

Please provide model checks demonstrating that the fitted switching model (with SPS-dependent parameters) reproduces the key summaries used in the manuscript (switch counts, state occupancy/time spent in each percept, and state-specific duration distributions), so that the DDM serves as an explanatory link rather than an additional layer of post hoc interpretation.

In the previous manuscript, we agree that the central concept of “perceptual stability” was not sufficiently defined, which could lead to apparent inconsistencies across analytic frameworks. In the revised manuscript, we therefore clarify that “stability” includes at least two components: (i) an overall bias/occupancy component (how much time is spent in a given state) and (ii) a state-maintenance component (how likely a state is to terminate once established). These two components may produce similar effects in the behavioral results, but they are conceptually separable. We hope that this new distinction clarifies the overall picture.

With respect to the requested model checks, we agree that such checks would have been extremely useful for the DDM framework. However, we felt than even such checks could not have compensated the gaps in the mapping from DDM to mechanistic interpretation, pointed out by all reviewers. Thus, as stated above, we removed the DDM analysis entirely.

Relatedly, the manuscript reports many outcomes and parameter-level associations across multiple analytic frameworks, which increases the risk of selective emphasis and inflated false positives even when some corrections are applied locally. It would help to clarify which outcome(s) are central to the main claim, and how the remaining analyses could be interpreted (supporting/exploratory). Please also state clearly how multiple testing was handled across these result families.

Indeed, the multiple-models approach, viewed as a strength by Reviewer #1, inevitably entails concerns about selective emphasis and multiplicity. We share these concerns and, in the revision, we tried to clarify which outcomes are crucial to our interpretations, and which are instead supporting analyses or robustness checks. In particular, the small sequential dependence effect is now reported as Supplementary Information. We also revised the organization of the Results to make this hierarchy explicit and to avoid over-emphasizing isolated parameter-level findings.

3. The Introduction places SPS/HSP within a broad "environmental sensitivity" framework and related literature, but the empirical contribution is narrowly centered on bistable auditory streaming dynamics and parameter interpretations within that paradigm. As currently written, the framing can read as "many traits covary with bistability, so we also tested HSP," rather than a hypothesis-driven link. Please tighten the narrative by 1) adding a more specific mechanistic prediction connecting SPS to auditory scene analysis/stream segregation, and 2) keeping broader claims about environmental

sensitivity proportional to what can be supported by a single bistable streaming task.

We completely agree that the Introduction should remain proportional to what can be supported by our data. In the revised manuscript, we tightened the narrative by briefly positioning SPS within environmental-sensitivity frameworks and then focusing on a specific prediction: as streaming has been shown to depend on a variety of acoustic cues, there was a reasonable possibility that it could be modulated by SPS. This is not highly specific, but hopefully, the new Discussion subsection “Predictive coding in auditory streaming and sensory processing sensitivity” now provides a possible specific link. As this was not an a priori hypothesis, we have refrained to present it as such in the Introduction.

4. The manuscript introduces "serial dependence" rather suddenly, in a way that can feel disconnected from the main modeling narrative and may be confusing given how strongly the term is associated with perceptual/decisional bias phenomena in the recent literature. In this paper, the relevant issue appears to be a methodological assumption: the models treat switches as independent events in a renewal process and the authors then test for sequential/history dependence in dominance durations (autocorrelations; parametric history kernels) and whether SPS moderates it. I recommend reframing the serial dependence section explicitly as a robustness check of the renewal/independence assumption (e.g., history dependence / sequential dependence of dominance durations, etc.), rather than presenting it under the banner of serial dependence, to reduce the risk of readers interpreting the section as addressing the broader serial-dependence literature.

We thank the reviewer for this helpful suggestion. We agree that the term “serial dependence” is often used in the recent literature to denote perceptual bias effects and can therefore be misleading in the present context. In our study, concerns about history effects are essentially methodological: our models treat phase terminations as approximately renewal-like events, and therefore we wished to evaluate whether dominance durations exhibited sequential dependence that violated the independence assumption.

Following the reviewer’s recommendation, we have reframed this section explicitly as a robustness check of the renewal/independence assumption (using “sequential dependence of dominance durations” terminology) rather than presenting it under the broader banner of serial dependence. We also moved the corresponding analyses to **Supplementary Information** and present them as supporting diagnostics, so that readers do not interpret this component as a contribution to the broader serial-dependence literature. The main conclusions are unchanged, and the robustness check provides additional reassurance that the reported SPS associations are not an artifact of short-range sequential dependence.

5. Alternative explanations linked to attention and response strategy need to be addressed more

directly. With long continuous reporting, lower switch rates can reflect conservative criteria, delayed reporting, fatigue, or compliance differences rather than perceptual dynamics per se, and SPS may correlate with such factors as well as with anxiety traits, musical experience, or auditory acuity. Please expand the limitations accordingly and, if any relevant measures are available, report them; if not, consider simple robustness checks to rule out the most obvious reporting-strategy artifacts (e.g., participants with extremely low switch counts or evidence of prolonged unresponsive periods).

We agree that, in long continuous-report paradigms, reduced switch rates and longer reported dominance durations could reflect non-perceptual factors such as the ones you describe. And indeed, SPS may covary with these non-perceptual factors. Unfortunately, we did not collect measures of the potential covariates you mention (anxiety traits, musical experience, or auditory acuity), so we cannot test them directly. We therefore expanded the Limitations section to state this explicitly (see also our response to the major comment 1 of Reviewer #1). We also note that, as our main effect was highly specific and in particular, perceptual-state dependent, it is not immediately clear how it could be explained by non-perceptual factors.

In addition, we would like to point out that we took steps to reduce the influence of the most obvious reporting artifacts and to keep inference conservative. Specifically, we applied a preprocessing rule that removes very short segments (< 0.5 s) likely reflecting report noise and excludes unusually long phases (> 30 s) that are more plausibly driven by lapses in continuous reporting than by perceptual dynamics. In the revised manuscript, we also centered our primary inference on Bayesian model-based estimates, which quantify uncertainty directly and reduce reliance on any single extreme observation. These revisions aim to keep the claims operational and to make clear which interpretations are supported by the present data and which require future work with additional measurements of potential covariates.

Minor

1. The hazard-model description is inconsistent with respect to temporal binning and the reported quantity. In different parts of the manuscript the hazard is described in incompatible units (e.g., 100-ms bins vs 1-s intervals), which makes it difficult to reproduce the analysis as written. Please state the bin width consistently, define whether the dependent variable is a per-bin event probability or a rate, and describe the time basis functions/smoothing and covariate coding in a way that matches what is plotted and reported.

Thank you very much for spotting this inconsistency. In the revised manuscript, we have standardized the discrete-time hazard analysis to 100-ms bins throughout and revised the text to use consistent terminology. Specifically, the dependent variable is defined as a per-bin termination event probability, rather than a continuous-time rate. We now state the bin width consistently wherever the hazard model is described and where results are reported and plotted.

We also expanded the Methods to align the written description with the implementation and the plotted quantities: (i) the exact time basis used to model the elapsed-time dependence of termination probability, (ii) covariate coding for SPS and perceptual state (including the interaction term), and (iii) the random-effects structure. Finally, to facilitate full reproducibility, we provided the analysis scripts and model inputs in the OSF repository referenced in the Data and Code Availability section.

Relatedly, in Fig. 4, the shaded regions are not clearly defined in the legend (e.g., whether they represent bin widths, uncertainty bands, or highlighted evaluation windows). As currently presented, the shading can be misread as indicating a continuous time interval over which the hazard difference is significant, even with the additional "ns" labels. Please explicitly describe what the shaded regions represent in the legend, and align the visualization with the discrete-time/binning and selected evaluation-time testing procedure (e.g., clarify that significance is assessed only at the pre-specified time points with Holm adjustment).

Following your helpful suggestion, we have aligned the visualization with the discrete-time analysis and the planned evaluation procedure. Specifically, in the updated hazard figure (now **Figure 3**), the only shaded bands are 95% credible intervals around the posterior hazard estimates, as stated explicitly in the caption, and they do not denote evaluation windows or "significant periods."

To avoid any interpretation that significance is assessed over a continuous time interval, we report inferential comparisons only at prespecified time points via planned posterior contrasts (**Supplementary Table 1**). We describe these discrete-time-point results in the main text. We also revised the Methods to define the hazard consistently as a per-bin termination probability in 100-ms bins and to make the evaluation procedure explicit.

2. Please add any missing methodological details needed for replication, including audio presentation details (calibration/level, device/headphones, sampling rate, etc.), and clarify instructions about how participants should report during ambiguous or transitional percepts.

We apologize for the omissions. The Methods already specified the testing environment, headphone model (Sennheiser HD 599), stimulus delivery mode (diotic), and the continuous key-hold reporting procedure implemented in Presentation (now p. 20). But to further improve reproducibility, we have now added the missing technical details as requested, including the response-recording sampling rate and the audio calibration information. We also clarified the important point about reporting instruction for ambiguous or transitional percepts, stating how participants were asked to respond when their percept was uncertain or changing.

3. The statement "In an FPT framework, by contrast, a switch is described as the completion of a small

number of discrete sub-events" (ll. 255–258) is misleading. A standard DDM is itself an FPT model (diffusion-to-bound), and the manuscript elsewhere contrasts diffusion-to-bound (inverse-Gaussian waiting times) with discrete sub-event accumulation (Gamma/Erlang). Please revise the phrasing to reflect that the FPT perspective is a broader umbrella (including diffusion-to-bound/DDM) and that the discrete sub-event account is one candidate mechanism within that perspective, rather than its definition.

Also, the phrasing "each switch is treated as continuous accumulation" is imprecise. In a DDM, the switch is a boundary-crossing (first-passage) event, whereas the continuous accumulation describes the latent process during the interval between switches.

We thank the reviewer for this important clarification. We have revised the wording accordingly and removed the imprecise "continuous accumulation" phrasing, making clear that the accumulation refers to the latent process during the interval between switches and that the switch itself is a first-passage (criterion-crossing) event.

As the DDM analysis has been removed from the manuscript, the FPT language is now used only to motivate the Gamma/Erlang distributional decomposition and is stated in a way that does not imply that FPT is synonymous with discrete sub-events.

Reviewer #3

Summary

The authors used a number of modeling approaches to estimate the impact of Sensory Processing Sensitivity (SPS), per the Highly Sensitive Person (HSP) scale, on auditory stream processing. Their paradigm showed a balanced bistable effect, whereby participants reported hearing one and two streams with relatively equal probability. Individuals with higher SPS perceived auditory streams for longer durations, with fewer switches between streams and greater stability (in the 8-20s window after percept onset), than lower SPS individuals. Additionally, HSP scores were associated with a tendency towards perceiving segregated streams, and that switching from segregated to integrated streams involved fewer, noisier decision steps.

We thank the reviewer for the clear and accurate summary of our main findings.

Major Feedback

Overall, the paper tests an interesting question about the interaction of external stimuli and individual traits that influence endogenous processing, and discusses the use of models in future research, thus adding to the field. However, the paper is highly technical, with several core concepts not fully defined, limiting the paper's accessibility. Consider explaining concepts such as exploration (in the context of ego-resiliency), sub-events, direction-specific entry, for readers not in your immediate field and use

more plain language in your initial summary of the results in the Discussion.

We appreciate the reviewer's point about accessibility. In response, we revised the manuscript to reduce technical load and improve clarity for non-specialist readers. Specifically, we simplified the initial results summary in the Discussion and clarified terminology at first mention. Removing the DDM analysis (see below) also helped streamlining the argument. We also addressed the reviewer's specific concern about "exploration" in the context of ego-resiliency by revising the relevant framing in the Introduction.

The authors mention the models are meant to be complementary, but there seem to be a number of measures capturing aspects of switch propensity that are likely related and sharing variance. The paper would benefit from additional discussion of what potential mechanisms might explain the HSP effects on drift and hazard but not k.

We fully agree that several of the descriptive summaries of switching behavior are related and likely share variance, as you point out. In the revision, we therefore clarified the presentation by decomposing "perceptual stability", as observed phenomenologically in the behavioral data, into conceptually separable components, namely overall state occupancy versus state maintenance. Both could impact switch propensity, as you phrase it. Moreover, we now focused the primary inference on model-based estimates of maintenance, rather than treating multiple correlated summaries as independent evidence.

Regarding the distributional decomposition, our results suggest that SPS is more consistently modulating the timescale component of dominance durations than in the shape/regularity parameter. We interpret this pattern cautiously: it is consistent with SPS modulating how long states tend to persist without strong evidence for a systematic change in the regularity/heavy-tail of the duration distribution. We now present this as a descriptive implication of the decomposition rather than as definitive support for a specific mechanistic account. The hypothetical mechanistic account, still highly speculative, is now limited to the Discussion subsection "Predictive coding for auditory streaming and sensory processing sensitivity."

I appreciate the discussion around serial dependencies and independence assumptions in bistable perception models, yet I wonder about the DDM implementation. While HSP doesn't modulate autocorrelation, autocorrelation still exists. Why not use an autoregressive (AR1) DDM structure? Additionally, was the interaction between switch direction and autocorrelation tested?

We thank the reviewer for this suggestion. We agree that temporal dependence is an important concern in bistable-perception data, which would have been interesting to test in the way suggested (AR1 extension) in the DDM framework. However, in the revised manuscript, we removed the DDM analysis entirely in response to broader concerns about the appropriateness and interpretability of

mapping its parameters on the continuous bistable switching task.

Instead, we explicitly treat sequential dependence as a methodological robustness check of the renewal/independence assumption. We report these diagnostics in **Supplementary Information** and clarify that, while short-range dependence can be present, there is no evidence that SPS reliably moderates these history effects. This framing ensures that the main conclusions about SPS-related state maintenance are not contaminated by short-range dependence.

Minor Feedback

- Consider more clearly introducing ABA-tone sequence, for those unfamiliar (e.g. "the classic sequence of triple tones, ABA, listeners …")

We have revised the Introduction to introduce the ABA triplet paradigm more explicitly.

- Line 68-9, do authors mean to write "to treat switches AS perceptual decisions"?

Thank you for catching this. We corrected the wording and revised the sentence for clarity, avoiding decision-task terminology in the revised manuscript.

- Fig 3 caption, Line 192, very minor but it would read more smoothly to give a little more context in the caption where it reads (B) As (A) (e.g. (B) As above, or Similar to panel (A))

Thank you. The figure referred to in the previous version has been removed during the revision and the figure set has been reorganized accordingly, so this caption issue no longer applies.

- Fig. 4 should have a legend for the different lines, not just written in the caption.

We agree and have revised the figure accordingly. In the revised manuscript, the hazard figure (now **Figure 3**) includes an explicit legend identifying the curves corresponding to the three SPS levels (HSP score: −1 SD, mean, +1 SD), and the caption specifies that shaded bands represent 95% credible intervals.
